# Supplementary material for: Adverse Outcome Pathways Associated with the Ingestion of Titanium Dioxide Nanoparticles—A Systematic Review
Source: Nanomaterials (Basel). 2022 Sep 21;12(19):3275. doi: 10.3390/nano12193275 (PMC9565478; doi:10.3390/nano12193275)
Supplement: Supplementary file 1 [file nanomaterials-12-03275-s001.zip › Table S2.pdf]

Table S2 – Studies reporting the cellular and systemic uptake of TiO2-NPs *in vitro* and *in vivo*.

Reference	Study description	Main findings concerning cellular uptake
Ruiz et al., 2017 [36]	*In vitro* and *in vivo* exposure.<br><br>Assessment of the effects of food derived TiO2 (rutile, 25-50 nm) in cultured human intestinal epithelial cells and macrophages (THP-1 cells), as well as in GIT cells (Caco-2, and HT-29 cells). The ability of TiO2 to traverse epithelial monolayers and accumulate in the blood of patients with Inflammatory bowel disease, was assessed using inductively coupled plasma mass spectrometry (ICP-MS).	● TiO2 particles were shown to disturb epithelial barrier function in Caco-2 monolayers.<br>● A dose-dependent accumulation in mice spleen, pointing to the fact that ingested TiO2 can traverse the intestinal mucosa and reach the systemic circulation.
Song et al., 2015 [44]	*In vitro* exposure.<br><br>In this study, the cytotoxicity and translocation of two native TiO2 NPs, and these two TiO2 NPs pretreated with the digestion simulation fluid or bovine serum albumin were investigated in undifferentiated Caco-2 cells, differentiated Caco-2 cells and Caco-2 monolayer. Flow cytometric light scatter analysis and TEM were used to evaluate uptake.	● Undifferentiated Caco-2 cells swallowed native TiO2 NPs easily, but not pretreated NPs (protein coating on NPs impeded the cellular uptake).<br>● Compared with undifferentiated cells, differentiated ones possessed much lower uptake ability of these TiO2 NPs.<br>● The traverse of TiO2 NPs through the Caco-2 monolayer was also negligible.
Pedata et al., 2019 [37]	*In vitro* exposure.<br><br>Conceivable mechanisms by which nano-sized TiO2 particles affect physiological function of the intestinal epithelium layer were analyzed using the differentiated Caco-2 cell line as a model of the human intestinal mucosa to identify the biological response triggered by TiO2 particles. Transport and ultrastructural localization of TiO2 nanoparticles were determined by ICP-OES, TEM and ESI/EELS analysis, respectively.	● TiO2 NPs were internalized and preferentially entrapped by Caco-2 monolayers, with detrimental effects on paracellular permeability<br>● Exposure to 42 mu g/mL TiO2 nanoparticles disrupted the tight junctions-permeability barrier with a prompt effect detectable after 4h incubation time and wide effects on barrier integrity at 24 h.<br>● Storage of TiO2 NPs inside the cells affected enterocytes viability.
MacNicoll et al., 2015 [47]	*In vitro* and *in vivo* exposure.<br><br>This study investigated the uptake and biodistribution of nano-and larger-sized TIO2 using an in vitro model of gut epithelium (Caco-2 cells) and *in vivo* in rat. Internalization was evaluated by sp-ICP-MS.	● TiO2 NPs did not lead to any significant translocation either to blood, urine or to various organs in rat at any of the time intervals studied over a 96 h post-administration period<br>● Different methods used for dispersing particles did not affect the uptake, and orally administered TiO2 was found excreted in the feces over a period of time.
Koeneman et al., 2010 [31]	*In vitro* exposure.	● TiO2 was able to penetrate into and through the cells without disrupting junctional complexes

	Both toxicity and mechanistic studies were employed to investigate possible pathways by which TiO_2 nanoparticles could cross the epithelium layer. To monitor Caco-2 cell epithelial integrity, TEER was employed.	● At 10 µg/mL and above of TiO_2 nanoparticles cross the epithelial lining of the intestinal model by transcytosis, albeit at low levels, and begin alteration of both microvillar organization on the apical surface of the epithelium.<br>● When 1,000 µg/mL TiO_2 was applied for a short time (24 h), it appears the microvilli were being absorbed into the cell.
Urrutia-Ortega et al., 2016 [7]	*In vitro* and *in vivo* exposure.<br>Study focused on the oral E171 intake impact on the enhancement of colorectal tumor formation in a colitis associated cancer mice model.	● E171 group showed an increase of crypts size and number, revisited with hyperplastic epithelium with slight dysplastic changes<br>● Goblet cells in the colon decreased (that act as protection barrier in colon)
Meyer et al., 2019 [43]	*In vitro* exposure.<br>Visualization and quantification of cellular NM uptake as well as their interactions with biomolecules within Caco-2 cells was assessed. A combination of the label-free spatially resolved dosimetric tools, micro resolved particle induced X-ray emission and Rutherford backscattering, together with high resolution imaging techniques, such as TOF-MS and TEM, were applied to visualize the cellular translocation pattern of TiO_2 NMs and to quantify the NM-load, cellular major, and trace elements in differentiated Caco-2 cells as a function of their surface properties at the single cell level.	● Internalized NMs are not only able to impair the cellular homeostasis by themselves, but also to induce an intracellular redistribution of metabolically relevant elements such as phosphorus, sulfur, iron, and copper.<br>● TiO_2 load across a cell population suggested endocytosis as the uptake mechanism.<br>● The cellular NM load was highly dependent on exposure conditions for the hydrophobic NMs, with uptake efficiency up to 14% of the administered NMs.<br>● In case of the hydrophilic NMs, the uptake efficiency was around 3%, independent from the exposure scenario.
Ammendolia et al., 2017 [19]	*In vitro* and *in vivo* exposure.<br>This study investigated potential modulatory effects of low doses ofTiO_2 NPs on intestinal cells in vivo in rats and in vitro (HT-29 cells). ICP-MS and TEM were used to evaluate TiO_2 cellular internalization.	● After short-term exposure to TiO_2 nanoparticles in rats, histopathological analysis of intestinal tissues indicated a gender-specific effect with increased length of intestinal villi in male rats only.<br>● Intestinal tissue showed nanoparticle deposition
Mohammadipour et al., 2014 [130]	*In vivo* exposure.<br>This study aimed to determine the effects of exposure to TiO_2-NPs during pregnancy on hippocampal cell proliferation and the learning and memory of offspring. Pregnant Wistar rats received intragastric TiO_2-NPs.	● TiO_2 NPs were accumulated in the hippocampus of rats<br>● Cell proliferation, memory, and learning are affected by developmental exposure to these nanoparticles

Shrestha et al., 2016 [120]	Ex- vivo oral exposure.<br><br>Four kinds of TiO2 nanorods (TiO2 NRs), with different surface functional groups, were used to study their interaction with rat bone marrow stem cells (MSCs). The cellular uptake of the TiO2 NRs was qualitatively studied by TEM and then quantified by ICP-MS.	● The results showed that the MSCs ingested larger amounts of TiO2-core NRs and TiO2-NH2 NRs than those of TiO2-COOH NRs and TiO2-PEG NRs, with similar intracellular distribution patterns
Hu et al., 2018 [110]	*In vivo* oral exposure.<br><br>In this study, mice were challenged with TiO2 NPs in order to add knowledge about whole-genome effects of ingested TiO2 NPs as xenobiotics, at 50 mg/kg b.w. ICP-OES was used for TiO2 detection.	● Titanium concentrations were increased in the liver, pancreas, spleen, kidney, and small intestine of the mice in all TiO2 NPs groups<br>● TiO2 NPs impact on genome-wide pathways, especially the plasma glucose homeostasis-related pathways in mice orally administered with 10-200mg/kg b.w. of TiO_2
Konstantinova et al., 2017 [216]	*In vitro* and *ex-vivo* exposure.<br><br>Reconstructed normal human buccal mucosa was generated from primary normal human oral keratinocytes and fibroblasts isolated from buccal oral mucosa of healthy patients. The reconstructed tissues were exposed after 10 days to clinically relevant concentrations of spherical or spindle rutile nano-TiO2 in suspension for short (20 min) and longer time (24 h). Ultrahigh-resolution imaging (URI) microscopy was used to assess the depth of penetration into reconstructed tissues.	● Tissue architecture and microanatomy was not altered by exposure to nano-TiO2<br>● Para-cellular way of transport nano-TiO2 penetrated a reconstituted human normal buccal epithelium very soon after exposure<br>● The penetration was shape-, dose- and time-dependent, and most of the particles remained within the upper third of the epithelial tissue<br>● The depth of penetration diminished in time at higher concentrations
Tay et al., 2014 [55]	*In vitro* exposure.<br><br>The cellular internalization of TiO2-NPs was characterized using TR146 human oral buccal epithelial cells as an *in vitro* model.	● TiO2-NPs were able to bind to the cellular membrane and pass into the cells in a dose dependent manner
Nejad et al., 2016 [118]	*In vitro* exposure.<br><br>Effects of high intensity focused ultrasound (HIFU) combined with photocatalytic TiO_2-NPs as semiconductor, were investigated in human oral squamous cell line HSC-2, used as cancer therapy.	● Penetration of TiO2 nanoparticles into the cell cytoplasm was only observed in HIFU + TiO2 treated tissues
Faust et al., 2014 [38]	*In vitro* exposure.	● Food-grade TiO2 at 350 ng/mL (100 ng/cm(2) cell surface area), disrupted the normal organization of the microvilli and ~42% loss of intestinal brush border surface

	Using a combination of scanning and transmission electron microscopy it was studied the biological response to food grade TiO2 exposure on the Caco-2(BBe1) cell system.	● Loss of microvilli from the Caco-2(BBe1) cell system was due to a biological response, and not simply a physical artifact of *in vitro* exposure.
Jalili et al., 2018 [86]	*In vitro* exposure.<br><br>The aim of this study was to investigate the cytotoxic and genotoxic effects of two rutile TiO2 NMs (NM103 and NM104), on intestinal (differentiated CACO-2) and hepatic cell (differentiated HEPARG) models, following 3 or 24 h treatments with concentrations of TiO2 NMs from 1.2 to 80 μg/cm^2. TEM imaging was used to detect TiO2.	● The presence of the two TiO2 NMs within the cytoplasm was observed.
Garcia-Contreras et al., 2014 [123]	*In vitro* exposure.<br><br>Evaluation of the effect of TiO2 NPs on the drug-sensitivity of oral squamous cell carcinoma and inflammation of human gingival fibroblasts (HGFs). Intracellular uptake and distribution of TiO2 NPs were assessed by TEM.	● TiO2 NPs clusters were dose-dependently incorporated into the vacuoles of cells.
Teubl et al., 2015 [124]	*In vitro* and *ex vivo* exposure.<br><br>Interactions of three TiO2 NPs (NM 100, NM 101 and NM 105) with oral tissues were investigated. Physicochemical properties, intracellular localization and potential toxic effects were evaluated in in vitro buccal epithelial TR146. Particle penetration was investigated with an *ex vivo* model using porcine mucosa. Multiphoton microscopy was introduced as an accurate method to detect TiO2 particles within the tissue.	● Differences in the penetration depth were observed depending on the particle characteristics. NM 100 and NM 105 were found in both the upper part and the lower part of the buccal mucosa, while NM 101 (smallest particle sizes) only penetrated the upper parts.<br>● Transport studies revealed that TiO2 nanoparticles were found in vesicles, as well as freely distributed in the cytoplasm.
Gitrowski et al., 2014 [217]	*In vitro* exposure.<br><br>The gastrointestinal uptake of different crystal structures of TiO2 was investigated using Caco-2 intestinal cells. TiO2 was detected by EDS.	● Electron micrographs of the Caco-2 monolayer showed the presence of Ti particles inside the cells, in all the studied exposures.<br>● Caco-2 monolayers exhibited time-dependent, saturable uptake of Ti from TiO2 exposures, which was influenced by crystal type.<br>● Ti accumulation for TiO2 NP exposure in Caco-2 cells is crystal structure-dependent, and the mechanism(s) involves endocytosis of intact particles.

Bettini et al., 2017 [3]	*In vitro* and *in vivo* exposure.<br><br>Chemically induced colon carcinogenesis rat model was orally exposed for one week to E171 at human relevant levels or NM 105. Intracellular uptake of TiO2 NPs was assessed by TEM.	● Titanium was detected in the immune cells of Peyer's patches (PP) as observed with the TiO2-NP model NM-105.<br>● A 100-day E171 treatment promoted colon microinflammation and initiated preneoplastic lesions while also fostering the growth of aberrant crypt foci in a chemically induced carcinogenesis model
Dorier et al., 2017 [81]	*In vitro* exposure.<br><br>Exposure to E171 and two different types of TiO_2-NPs, either acutely (6-48 h) or repeatedly (three times a week for 3 weeks, using two in vitro models: (i) a monoculture of differentiated Caco-2 cells and (ii) a coculture of Caco-2 with HT29-MTX mucus-secreting cells. ICP-MS was used to evaluate Ti content.	● E171 damaged Caco-2 and HT29-MTX cells<br>● E171 effects were less intense after acute exposure compared to repeated exposure, which correlated with higher Ti accumulation.<br>● The effects were also more intense in cells exposed to E171 than in cells exposed to TiO2-NPs.
Bu et al., 2010 [40]	*In vivo* exposure.<br><br>In the present study, the oral toxicological effects of TiO2 NPs were investigated using conventional approaches and metabonomic analysis in Wistar rats. Serum chemistry, hematology and histopathology examinations were performed. Nuclear magnetic resonance (NMR) was used for TiO2 detection.	● Disturbances in energy and amino acid metabolism and the gut microflora environment may be attributable to the slight injury to the liver and heart caused by TiO2 NPs.<br>● Alteration on membrane permeability and loss of membrane integrity was observed.
Talbot et al., 2018 [42]	*In vitro* and *in vivo* exposure.<br><br>A comprehensive approach was performed to evaluate in vitro and in vivo interactions between TiO2 and intestinal mucus, by comparing food-grade E171 with NM-105. E171-trapping properties of mucus were analyzed *in vitro* using HT29-MTX intestinal epithelial cells. It was also compared TiO2-mediated effects *in vivo* in rats after acute or sub-chronic oral daily administration of food-grade E171 and NM-105 at relevant exposure levels for humans. Time-lapse confocal laser scanning microscopy was performed and the penetration of E171 TiO2 into the mucoid area of HT29-MTX cells was visualized *in situ*.	● By confocal observation, one hour after exposure TiO2 accumulation seems to be trapped into mucus.<br>● Food-grade TiO2 is trapped by intestinal mucus *in vitro* but does not affect mucin O-glycosylation and short-chain fatty acid synthesis *in vivo*, suggesting the absence of a mucus barrier impairment under healthy gut conditions.
Natarajan et al, 2015 [119]	*In vitro* and *Ex-vivo* exposure.<br><br>Primary rat hepatocytes were exposed to three different TiO_2 NPs (commercially available rutile, anatase and P25) and cells were evaluated for hepatocyte functions and	● All three TiO2 nanoparticles induced significant damage in hepatocyte functions, even at concentrations as low as 50 ppm with commercially used P25 causing maximum damage.

	mitochondrial dynamics on primary rat hepatocytes. Cellular morphology was observed by SEM.	
Richter et al, 2018 [39]	*In vitro* and *in vivo* exposure.<br><br>The interaction between TiO_2 NMs exposure, beneficial bacterial populations (Lactobacillus rhamnosus GG), and intestinal function was investigated in co-cultures of Caco-2 and HT29-MTX. The *in vitro* model, experiments were performed with *D. melanogaster* which has a gut much like the human gut. Glucose uptake and transport in the presence of TiO2 nanoparticles was assessed using a fluorescent glucose analog.	● Cell monolayers exposed to physiologically relevant doses of TiO2, resulted in a statistically significant reduction in glucose transport.<br>● The decrease in glucose absorption was caused by damage to intestinal microvilli, which decreased the surface area available for absorption.<br>● Damage to microvilli was ameliorated in the presence of L. rhamnosus.<br>● Studies in *Drosophila melanogaster* showed that TiO2 ingestion resulted in decreased body size and glucose content.
Pele et al., 2015 [218]	*In vivo* Human exposure<br><br>In this study, pharmaceutical/food grade TiO2 absorption was evaluated after human volunteers' ingestion (with normal intestinal permeability). Humans were orally administered with 100 mg pharmaceutical/food grade TiO2. Blood samples were collected from 0.5 to 10 h post ingestion and analyzed for the presence of reflectant bodies (particles) by dark field microscopy, and for total titanium by ICP-MS.	● A fraction of pharmaceutical/food grade titanium dioxide is absorbed systemically by humans following ingestion.<br>● TiO2 is directly absorbed into the bloodstream of healthy volunteers.<br>● Direct uptake and persistence occurs after 2h and 6h ingestion, one in early in the proximal small intestine and one late in the distal small intestine.
Winter et al., 2011 [106]	*In vitro* exposure.<br><br>The objective was to compare the *in vitro* effects of micron-sized Silica with nano-sized TiO2 particles (5, 20 and 40 mg/cm^2) on intestinal dendritic cells (DC) derived from bone marrow of C57BL/6 mice. Uptake of particles by DC was evaluated by means of flow-cytometry.	● Nanoparticles used in this current study preferably enter DC via actin-dependent mechanism.<br>● TiO2 and SiO2 nanoparticles uptake via actin-dependent mechanisms is required for the activation of the inflammasome.
Schneider et al., 2017 [107]	*In vitro* exposure.<br><br>Toxicological behavior of metal nanoparticles (gold, silver) and metal oxide nanoparticles (copper oxide, zinc oxide, titanium dioxide) *in vitro* in human colorectal adenocarcinoma cells (HT29), after 24-h nanoparticle treatment with concentrations between 2 and 10 mu g/ml. Cellular uptake was analyzed by ICP-MS and TEM.	● It showed the incorporation of nanomaterials in HT29 human cells.<br>● The amount of metal taken up by a single HT29 cell, ranged from 0.02 pg/cell up to 1.39 pg/cell.

Martins et al., 2017 [59]	*In vivo* oral exposure.<br><br>The redox parameters and genotoxic effects in male Wistar rats treated with either AgNP or TiO_2 NP (0.5 mg/kg/day) individually, as well as under a co-exposure scenario, for 45 days, were evaluated. Tissue biodistribution of Ag and Ti was measured by ICP-MS.	● The tissue concentrations of Ti followed the order: kidneys > liver > blood.
Hu et al, 2016 [60]	*In vivo* oral exposure.<br><br>This study explored the effect of TiO_2 NPs in plasma glucose levels via ROS, after oral administration of 64 mg/kg bw/day of TiO_2 NPs for 18 weeks. Titanium content analysis in tissues was determined by ICP-OES and SEM.	● SEM results revealed that TiO2 NPs, and not their larger size aggregates, were found in liver and pancreas.
Liang et al., 2016 [126]	*In vitro* exposure.<br><br>Three engineered metal oxide nanoparticles (NPs) with similar size but significantly different solubility was used to study the cellular uptake, toxicity and immune activation of Raw 264.7 cells. Cellular internalization was evaluated by ICP-AES.	● TiO2 NPs only release very low concentration of ions.<br>● The cellular loading of the TiO2 NPs increased with the incubation time and Raw264.7 cells internalized the TiO2 NPs.
Jia et al, 2017 [98]	*In vivo* and *ex vivo* oral exposure.<br><br>This study evaluated the liver, brain, and embryo toxicity and the underlying mechanism of nano- TiO_2 after intraperitoneal exposure to 5, 10, 50 mg/kg/daily for 60 days, using mice models. TEM was used as a method to assess TiO2 NPs intracellularly.	● TiO2 NPs were distributed to and accumulated in the heart, brain, spleen, lung, and kidney of mice, in a dose-dependent manner.<br>● The organ/body weight ratios of the heart, spleen, and kidney were significantly increased, and those of the brain and lung were decreased.<br>● High doses of nano-TiO2 significantly damaged the functions of liver and kidney and glucose and lipid metabolism.<br>● *Ex vivo* mouse embryo models exhibited developmental and genetic toxicity after high doses of nano-TiO2.
De Angelis et al., 2013 [80]	*In vitro* exposure.<br><br>The toxicity (cell viability, uptake, ROS and inflammation) induced by zinc oxide (ZnO) and TiO2 NPs on Caco-2 cells was evaluated, after 6 and 24h exposure to 1-20 µg/cm2 (6.4–128.0 µg/mL). Titanium content in cells was measured by ICP-MS.	● Higher NPs cell interaction was observed for cells exposed to TiO2 NPs in serum-free culture medium than in complete cell culture medium.<br>● The higher cell interaction of TiO2 NPs observed, could be due to the spherical shape of these NPs.

Cai et al., 2019 [111]	*In vitro* exposure.<br><br>Determination of the effects related to the exposure route on the biodistribution, bio-persistence, and toxicology of nanoparticles (titanium dioxide, zinc oxide, and zirconium dioxide) in both mouse dams and their offspring. Oral and airway exposure routes were tested using gavage and intranasal administration, respectively. Biodistribution in the main organs (breast, liver, spleen, lung, kidney, intestine, and brain) and biopersistence in the blood and milk were determined by ICP-MS and TEM.	● A reduced offspring body weight was found with the reduced nanoparticle size.<br>● Both oral and airway exposure increased the nanoparticle concentrations in the main tissues and milk.
Murugadoss et al., 2020 [75]	*In vitro* and *in vivo* exposure.<br><br>Tested two anatase TiO_2 NPs with different primary sizes (17 and 117 nm) and prepared ad-hoc suspensions composed of small or large agglomerates with similar dispersion medium composition. For *in vitro* testing, human bronchial epithelial (HBE), colon epithelial (Caco2) and monocytic (THP-1) cell lines were exposed to these suspensions for 24h; In vivo, C57BL/6JRj mice were exposed via oropharyngeal aspiration or oral gavage to TiO_2 suspensions for 3 days.	● *In vitro* testing: differential responses were observed only in THP-1 cells, among which, agglomeration of TiO2 NPs influences their toxicity/biological responses and, large agglomerates do not appear less active than small agglomerates. In HBE and Caco2 cells, the biological effects did not vary.<br>● *In vivo*: Large agglomerates of TiO2 induced higher pulmonary responses in aspirated mice and blood DNA damage in gavaged mice compared to small agglomerates.<br>● Ti was detected in the lung after 3 d, depending on NPs size.
Brun et al., 2014 [41]	*In vivo*, in vitro and *ex vivo* exposure.<br><br>To evaluate by in vitro models of gut epithelium (Caco-2 cells), a co-culture of Caco-2 and HT29-MTX cells and a coculture of Caco-2 and RajiB cells, as well as *in vivo* studies the impact and translocation of NPs through the gut epithelium. TEM- EDX was used for internalization observation.	● *In vivo* and *ex vivo* assays, revealed that agglomerates of TiO2-NPs are translocated through both the gut epithelium. Still TiO2-NP transepithelial passage is low.<br>● NPs cause *ex vivo* and *in vivo* increase of paracellular permeability, which is correlated to down-regulation of the expression of genes encoding junction proteins.<br>● In the regular *in vitro* models, TiO2-NPs neither cross the epithelium via the transcellular route nor via the paracellular route.<br>● Disruption is observed in the *in vivo* and *ex vivo* models while it is not observed in the *in vitro* models.

McCracken et al., 2013 [99]	*In vivo* and *ex vivo* exposure.<br><br>TiO2-NPs (21 nm) behavior in simulated digestion media and their interaction with intestinal epithelial cell line C2BBe1, a clone of Caco-2 cells. TEM was used to assess internalization.	● C2BBe1 cells internalized all TiO2-NPs tested.
Canli et al., 2020 [62]	*In vivo* oral exposure.<br><br>Effects of TiO_2 NPs in female rats following oral administration of differing doses (0, 0.5, 5, and 50mg/kg b.w./d) for 14 days. The images of the tissues were obtained using TEM to demonstrate TiO2 NP accumulation.	● TEM images demonstrated that TiO2 NPs accumulated in a dose-dependent manner in the tissues, suggesting translocation of NP from the intestine in all tissues.<br>● Data emphasized that the brain was the most sensitive organ against the effects of TiO2 NPs.
Sohal et al., 2020 [34]	In vitro exposure.<br><br>Five food-grade nanomaterials (TiO2, SiO2, ZnO, and two types of Fe2O3) were administered to an in vitro mucus-secreting co-culture (C2BBe1-Caco-2 subclone/HT29-MTX), to a triculture (C2BBe1/HT29-MTX/Raji B) and to a C2BBe1 (enterocyte) monoculture system to assess effects of exposure to each tested NM. Cell effects observations after challenge, were based on TEM, and TEER.	● Barrier integrity was less vulnerable to NMs exposures in tricultures than in monocultures<br>● TEER in tricultures was unaffected by any TiO2 exposures, but were reduced in monocultures by up to 50%<br>● morphological changes were observed in the brush border (blunted microvilli) in triculture assays;<br>● loss of cell viability and metabolic activity in cocultures but not monocultures at low TiO2 doses;<br>● increased membrane damage
Veronesi et al., 2012 [48]	In vitro exposure.<br><br>A digestion model was developed to test rutile TiO2 NPs (21 nm), during 48h at 50 lg/ml. The model of gastrointestinal epithelium was used using a co-culture of the subclone 1 of Caco-2 cells and Raji-B lymphocytes, which triggers the conversion of 50% of enterocytes into M-cells. Micro-beam x-ray absorption fine structure spectroscopy was used to investigate rutile TiO2 nanoparticles.	● Nanoparticles tend to accumulate into cells after 48 h exposure; however, no spectral differences arise between particles in cellular and in acellular environments<br>● No modification of the lattice properties of the nanoparticles occurs upon interaction with the barrier.

Farcal et al., 2015 [32]	*In vitro* exposure.<br><br>Six oxide NMs were tested in different laboratories using 12 cellular models representing 6 different target organs/systems (immune system, respiratory system, gastrointestinal system, reproductive organs, kidney and embryonic tissues), applying 10 different assays (cytotoxicity, embryotoxicity, epithelial integrity, cytokine secretion and oxidative stress. The primary particle size for both NM-103 and NM-104 is in the range 22–26 nm ± 10 nm measured by TEM. TEER was used as an indicator of epithelial cell barrier integrity.	● Longer term exposure (7 to 21 days) did not significantly affected the cell barrier integrity in the presence of TiO2<br>● A cell-specific response to NMs was observed in the present study.<br>● Upon short term exposure (24-72h), immune cells showed a higher sensitivity to NMs, followed by testicular cells and airway epithelial cells.
Hendrickson et al., 2016 [63]	*In vivo* oral exposure.<br><br>This study compares the biodistribution of titanium dioxide nanoparticles of different sizes (5-10 nm and 20-25 nm) after peroral administration to rats. Inside the different studied organs, Titanium was detected by atomic absorption spectroscopy (AAS).	● The absorption from the gastrointestinal tract (GIT) and translocation into secondary organs of titanium dioxide occurred in a size-dependent manner.<br>● The smaller nanoparticles were found in the brain, lungs, heart, liver, kidneys, spleen, small intestine, testicles, and blood<br>● The larger nanoparticles were accumulated only in the liver, kidneys, spleen, and small intestine<br>● The amounts of the detected titanium were much smaller than the administered doses.
Yang et al., 2017 [56]	*In vivo* oral exposure.<br><br>In this study, after mice were orally administered TiO2 NP (21 nm) for 14 days, the serum and liver tissues were assayed by biochemical analysis, real time quantitative polymerase chain reaction, western blot and TEM.	● Deposition of TiO2 NP in hepatocytes and the abnormality of microstructures was observed.<br>● Oral administration of TiO2 NP (21 nm) led to deposition of particles in hepatocytes, mitochondrial edema, and the disturbance of liver metabolism function.
Morgan et al., 2017 [151]	*In vivo* oral exposure.<br><br>The effect of anatase TiO_2 NMs (10 nm) was evaluated after 60 days of oral administration of 100 mg/kg b.w\day to albino Rat. The investigation was performed in order to assess the efficacy of tiron against TiO_2 NPs induced nephrotoxicity.	● It was observed renal pathological changes coupled with severe oxidative stress and fibrosis induced by long period of exposure to TiO_2 NPs<br>● Indications of over deposition of TiO_2 NPs in kidney tissue
Jones et al., 2015 [33]	Human volunteers and *in vitro* exposure	● There was not any drop in TEER values that might indicate damage or loss of cell junction integrity.

	The study was designed to conduct human in vivo and in vitro studies on the gastrointestinal absorption of nanoparticles, using titanium dioxide as a model compound, and to compare nanoparticle behavior with that of larger particles. Urine samples were collected over a 4-day period (24 h before dosing and 72 h post-dose), while blood samples were collected before dosing and at 2, 4, 24 and 48 h after dosing. Caco-2 cell line was used for the in vitro testing, and TEER was measured.<br><br>The extent of absorption, excretion or translocation of titanium dioxide particles was assessed by the determination of titanium using ICP–MS.	● The in vitro study demonstrated no evidence of titanium dioxide translocation across the cell layer into the receptor fluid<br>● extent of penetration into the basal chamber for all formulations was less than 0.05% of the applied dose<br>● TiO2NPs are likely to agglomerate in gastric fluid thus reducing a nanoparticulate dose during oral exposure.<br>● There is no evidence of significant absorption of titanium dioxide in humans after an oral dose, regardless of particle size.
Talamini et al., 2019 [58]	*In vivo* oral exposure.<br><br>This study investigated whether repeated oral administration of E171 to mice at a dose level (5 mg/kg body weight for 3 days/week for 3 weeks) comparable to estimated human dietary exposure, results in TiO_2 deposition in the digestive system and internal organs, and in molecular and cellular alterations associated with an inflammatory response.<br><br>NPs were analyzed by ICP-MS, DLS, Nanoparticle Tracking Analysis (NTA), and TEM.	● Repeated administration of food-grade TiO2 results in deposition in the GIT and the liver and is associated with molecular and cellular alterations in the inflammatory response
Onishchenko et al., 2012 [61]	*In vivo exposure.*<br><br>Penetration of titanium dioxide nanoparticles into enterocytes after their administration into an isolated loop of rat small intestine was studied *in vivo* by TEM. Using electron diffraction, titanium dioxide nanoparticles were studied in the apical regions of the cells.	● Titanium concentration in the liver increased significantly in rats receiving intragastrically rutile NP water dispersion in the high dose (100 mg/kg), which could indicate its penetration through the intestinal barrier.<br>● Uptake of TiO2 was not seen in other TiO2 forms
Jo et al., 2016 [45]	*In vivo* and *in vitro* exposure.<br><br>In the present study, in vivo solubility, oral absorption, tissue distribution, and excretion kinetics of food grade TiO2NPs were evaluated following a single-dose oral administration to rats and were compared to those of general grade TiO2NPs. The intestinal transport pathway was also assessed using 3-dimensional culture systems (with Caco-2 and RajiB co-cultures). Ti levels were evaluated by ICP-AES analysis.	● Absorptions of TiO2NPs were less than 0.8%, and most of the particles were directly eliminated through the feces, suggesting low toxicity potential of TiO2 NPs.<br>● The oral absorption of NPs considerably and significantly decreased when they were dispersed in distilled water, showing ~0.01% of absorption.<br>● Ti levels in the kidneys and lungs of rats significantly increased at 6 h.

Cabellos et al., 2017 [35]	*In vitro exposure.*<br><br>A Caco-2/Raji B co-culture was established to induce the transformation of enterocytes into M-cells. The model was characterized by monitoring TEER values and by SEM imaging. The translocation of TiO2 nanoparticles (NPs) was evaluated in the Caco-2/M-cell system and compared to that in a Caco-2 monolayer model.	● The exposure to TiO2 NPs did not cause changes in the morphology or TEER values of the cell monolayers.<br>● The levels of titanium recorded in the basal compartment were significantly higher than the background titanium levels only in the presence of M-cells.
Janer et al., 2014 [46]	*In vitro and in vivo exposure.*<br><br>In this study, different in vitro and in vivo test methods were used to evaluate cell uptake and oral absorption of TiO2 NPs. TEM and hydrodynamic diameters were measured by DLS using a Zetasizer (nano series Nano-ZS).	● TiO2NPs were readily uptaken by A549 cells in vitro.<br>● Very low translocation of TiO2NPs through a differentiated Caco-2 monolayer system<br>● Titanium levels in tissues did not increase after a single oral dose of TiO2NPs.<br>● Several NPs were observed in the cytoplasm of a cell from a Peyer's Patch by TEM.
