# Supplementary material for: Adverse Outcome Pathways Associated with the Ingestion of Titanium Dioxide Nanoparticles—A Systematic Review
Source: Nanomaterials (Basel). 2022 Sep 21;12(19):3275. doi: 10.3390/nano12193275 (PMC9565478; doi:10.3390/nano12193275)
Supplement: Supplementary file 1 [file nanomaterials-12-03275-s001.zip › Table S3.pdf]

Table S3 – Studies reporting the oxidative stress of TiO2-NPs *in vitro* and *in vivo*.

# Reference	Oxidative stress biomarkers	Study Description Model used	Main findings concerning ROS generation and oxidative stress
Jensen et al. 2019 [73]	*in vitro* -DCFH-DA; *in vivo* - human oxoguanine glycosylase 1 (hOGG1) +formamidopyrimidine-DNAglycosylase (Fpg)	Assessment of ROS generation in Caco-2 Cells and in mice liver and lung.	*in vitro* -No ROS generation in caco-2 cells *in vivo* - No changes in oxidative DNA repair activity in liver or lung.
Ruiz et al.2017 [36]	H2DCFDA	Assessment of ROS generation in Caco-2 Cells and HT-29.	Increase ROS generation following 30 min exposure to TiO_2, either in Caco-2 or HT29.
Proquin et al. 2017 [85]	ESR spectroscopy	*In vitro* exposure. E171 exposure of human Caco-2 and HCT116 cells to assess ROS generation and to investigate the contribution of the separate (MPs) and nano-sized (NPs) fractions to these effects.	Only MPs were capable of induced ROS formation in exposed Caco-2 cells.The capacity for ROS generation in a cell-free environment was highest for E171, followed by NPs and MPs. Neither E171 or TIO_2 NP induced ROS formation in exposed Caco-2 cells or HCT116 cells.
Gerloff et al. 2012 [87]	Total glutathione content HO-1 or γ-GCS oxidative markers (mRNA)	*In vitro* exposure. Five TiO_2-samples were analyzed for purity, crystallinity, primary particle size, SSA, zeta potential, and aggregation/agglomeration. The oxidative stress was evaluated in human intestinal Caco-2 cells.	No effects on ROS formation. No alteration in mRNA content of HO-1 or γ-GCS oxidative markers.
Song et al. 2015 [44]	DCFH-DA	*In vitro* exposure. In this study two native TiO_2 NPs, and two TiO_2 NPs pretreated with the digestion simulation fluid or bovine serum albumin were investigated in undifferentiated Caco-2 cells, differentiated Caco-2 cells and Caco-2 monolayer.	In undifferentiated Caco-2 cells the ROS generation was increased and concentration dependent in digested or not digested samples. No effect was observed in differentiated Caco-2 cells. ROS generation was not the main factor influencing the bio sequence of TiO_2 NPs in this study.

Brown et al., 2019 [88]	Total GSH content and Glutathione depletion assay; ToxTracker reporter assay - analysis of srxn1-GFP and Blvrb-GFP (Oxidative stress markers)	*In vitro* exposure. ToxTracker assay (mechanism-based reporter assay based on embryonic stem cells that uses GFP-tagged biomarkers for detection of DNA damage, oxidative stress and general cellular stress) used to evaluate food-grade TiO_2 (E171)) and compared to tissue relevant cell types. ROS was also assessed in Caco-2 and HepG2 cell lines.	E171 decreased total intracellular glutathione at the highest dose tested in HepG2 while no effect was observed in Caco-2 cells. The absence of Srxn1/Blvrb oxidative stress reporter activation suggests that E171 might induce low levels of ROS but these are not sufficient to induce an antioxidant response.
Canli et al, 2020 [62]	glutathione (GSH) and thiobarbituric acid reactive substances (TBARSs)	*In vivo* oral exposure. Effects of TiO_2 NPs in female rats following oral administration of differing doses (0, 0.5, 5, and 50mg/kg b.w./d) for 14 days. Oxidative stress was assessed by means of glutathione (GSH) and thiobarbituric acid reactive substances (TBARSs).	Levels of GSH forms did not change significantly, though there was a significant decrease in TBARS level at the highest NP dose.
Chen et al, 2019 [140]	Reduced glutathione (GSH); Oxidized glutathione (GSSG); Glutathione peroxidase (GSH-Px); Malondialdehyde (MDA); Superoxide dismutase (SOD)	*In vivo* oral exposure. To explore the gender susceptibility of TiO_2 NP-induced hepatic toxicity and the underlying mechanism in female and male Sprague-Dawley rats administered with TiO_2 NPs orally at doses of 0, 2, 10 and 50 mg/kg body weight per day for 90 days.	The changes of oxidative stress biomarkers indicated an oxidative stress state in the liver induced by subchronic oral exposure of TiO_2 NPs, which was gender dependent and the female rats seemed to be more susceptible than the male rats, to redox homeostasis. Indirect pathway to hepatic toxicity through oxidative stress and inflammatory responses.
Mohammed et al, 2019 [158]	MDA, GSH concentrations, and catalase activity in the liver.	*In vivo* oral exposure. The study aimed to demonstrate the influence of grape seed proanthocyanidin extract (GSE) on the hepatic TLR-4/ NF-κB signaling pathway in TiO2-NP-induced liver damage in male albino rats.	TiO2-NPs induced severe hepatic injury observed by biochemical markers, oxidative stress markers (increased MDA, decreased GSH, and catalase), and histopathological alterations.
Canli et al, 2017 [101]	total oxidant status (TOS); total antioxidant status (TAS), spectrophotometrically (Serum)	*In vivo* oral exposure. TiO_2 nanoparticles (NPs) were individually administered to female rats via oral gavage (0, 0.5, 5, and 50 mg/kg b.w./day) for 14 days.	Ti-NP increased the levels of all liver enzymes in the serum (up to 84%). The total oxidant levels in TiO_2-exposed rats were increased in the present study, but this was not statistically significant.

Tada-Oikawa et al., 2016 [83]	CM-H2DCFDA followed by flow cytometry	The present study was designed to determine the effects of five TiO2 particles of different crystal structures (anatase, rutile and mixture) and sizes in human epithelia colorectal adenocarcinoma (Caco-2) cells and THP-1 monocyte-derived macrophages.	Reactive oxygen species increased in both cell types, irrespective of the type of TiO_2, after 3h exposure to 25 and 50 µg/ml of TiO_2, although more pronounced in rutile forms.
Wang et al., 2013 [211]	reduced (GSH) and oxidized (GSSG) glutathione GSH/GSSG ratio glutathione peroxidase (GSH-Px)	*In vivo* oral exposure. A comparative toxicity study of TiO_2 NPs in 3-week (youth) and 8-week (adult) old Sprague-Dawley rats is reported following oral exposure at doses of 0, 10, 50, 200 mg kg − 1 body weight per day for 30 days.	Increased reduced glutathione (GSH)/oxidized glutathione (GSSG) ratios) in plasmas through enhancing the glucose and GSH levels in young rats or reducing the glutathione peroxidase (GSH-Px) activity and GSSG levels in adult rats.
Mohamed et al., 2015 [162]	Malondialdehyde (MDA) - lipid peroxidation; Nitric oxide (NO); Reduced glutathione (GSH); Catalase (CAT) activity.	*In vivo* oral exposure. To investigate the effects of nano TiO_2 on mice orally administered with 5, 50 or 500 mg/kg body weight for five consecutive days by oral gavage and sacrificed in different timepoints (24 hours or one or two weeks after the last treatment).	Significant persistent increase in malondialdehyde and nitric oxide levels and decreases in the reduced glutathione level and catalase activity compared with the control mice in a dose- and time-dependent manner.
Kermanizadeh et al, 2012 [220]	DCFH-DA total glutathione	*In vitro* exposure This study aimed to investigate the effects of five titanium dioxide (TiO_2) on oxidative responses in human hepatoblastoma C3A cell line as a model	ROS increased as measured by the DCFH-DA assay, but no changes in reduced GSH.. T
Chen et al., 2019 [154]	Glutathione; Glutathione peroxidase (GSH-Px), Lipid peroxidation products (malondialdehyde, MDA); Superoxide dismutase (SOD); Sulfhydryl groups (SH).	*In vivo* oral exposure. The effects of oral exposure to TiO_2 NPs was investigated on gut microbiota and gut associated metabolism in Sprague-Dawley rats administered with TiO_2 NPs (29 ± 9 nm) orally at population-related exposure doses (0, 2, 10, 50 mg kg−1), daily for 30 days.	The indirect pathway of oxidative stress and inflammatory response, probably due to dysbiosis of gut microbiota primarily (and increased production of lipopolysaccharides, LPS), played an important role in the mechanisms of toxicity induced by oral exposure to TiO_2 NPs. Increased MDA and decreased activity of SOD.

Orazizadeh et al., 2014 [79]	MDA ((TBARS), SOD and GPx	*In vivo* oral exposure. The aim of this study was to investigate the hepatoprotective effect of glycyrrhizic acid (GA) against hepatic injury induced by titanium dioxide nanoparticles (N TiO_2) in rats. N TiO_2-intoxicated rats received 300 mg/kg of TiO2 for 14 days by gavage method.	Increased lipid peroxidation<br>decreases SOD and GPx enzymes
Jafari et al., 2020 [221]	Superoxide dismutase (SOD), catalase (CAT), and glutathione peroxidase (GPx), malondialdehyde (MDA), ferric reducing antioxidant power (FRAP), reduced glutathione (GSH) levels	*In vivo* oral exposure. Twenty-four rats were orally exposed to TiO_2 (100 mg/kg BW/day), TiO_2 + thymol (10 mg/kg BW/day), and TiO2 + thymol (30 mg/kg BW/day), for 60 days.	Elevated MDA levels and reduction of antioxidant enzyme activities such as CAT, SOD, and GPx, and also FRAP and GSH levels in testis tissue.
Dorier et al., 2019 [201]	DCFH-DA;<br>mRNA expression of genes involved in ROS regulation: catalase (CAT), glutathione reductase (GSR), superoxide dismutase 1 and 2 (SOD1, SOD2).	*In vitro* exposure. The aim of this study was to document in vitro impact of the food additive E171, i.e. TiO_2 and TiO2 nanoparticles, on a co-culture of Caco-2 and HT29-MTX cells.	Increased intracellular ROS levels
Teubl et al., 2015 [96]	DHE (Dihydroethidium)	*In vitro* exposure. The ex-vivo permeability studies of TiO2 particles were performed with porcine buccal mucosa. This study addresses the biological impact of hydrophilic (NM 103, rutile, 20 nm) and hydrophobic (NM 104, rutile, 20 nm) TiO2particleswithin the buccal mucosa and HPRT gene mutation assay in V79 cells.	NM 104 particles showed a higher potential to decrease the physiological mitochondrial membrane potential than NM 103, resulting in a pronounced generation of reactive oxygen species.
Ammendolia et al, 2017 [19]	H2DCFDA	*In vitro* exposure. This study investigated potential modulatory effects of low doses of nano-sized titanium dioxide (TiO_2) on intestinal cells in vivo	Ros generation increased after 6h exposure to 2.5--20 μg/cm^2 concentrations. Ros generation increased after 24h exposure to 20 μg/cm^2 concentration. At 24 h interval time, DCHF fluorescence intensity appeared decreased compared to 6 h.

		and *in vitro* (HT-29). Oxidative stress was only studied in *vitro*.	
Hassanein et al., 2018 [100]	Lipid peroxidation (LPO)	*In vivo* oral exposure. The study aimed to assess the ameliorative effect of thymoquinone (TQ) on titanium dioxide nanoparticles (TiO_2NPs) induced acute toxicity in male rats.	Increased lipid peroxidation product (LPO) and decreased the level of antioxidants. Administration of TQ ameliorate the oxidative stress induced by TiO_2 NPs intoxication.
Cao et al., 2020 [91]	CellROX®	*In vitro* exposure. TiO_2 (E171) was incorporated into either a fasting food model (FFM) or a standardized food model (SFM). The food models, with or without TiO_2, were digested using a three phase GIT simulator, and the resulting digesta was applied to an in vitro small intestinal epithelial tri-culture cellular model for toxicity and ROS generation assessment. Micro-beam x-ray absorption fine structure spectroscopy was used to investigate rutile TiO_2 nanoparticles internalized into gastrointestinal cells during their crossing of a gut model barrier.	The difference in ROS production between FFM alone and untreated cells was negligible although increased for the highest dose. SFM alone increased ROS TiO_2 in SFM slightly increased ROS compared to SFM alone, suggesting that the effect of a more complex food matrix in this case was to eliminate the slight toxicity observed with TiO_2 in FFM.
Shrivastava et al., 2014 [222]	catalase - blood<br>glutathione (GSH) blood<br>glutathione peroxidase (GPx), - brain and liver<br>superoxide dismutase (SOD) - brain, liver and<br>blood<br>glutathione-S-transferase (GST). Brain and liver<br>(TBARS) - brain and liver<br>DCFH-DA assay in blood, liver	In vivo oral exposure. The study investigated the toxic effects of TiO_2 nanoparticles, on mouse erythrocytes, brain and liver after administered a daily oral dose of 500 mg/kg for 21 consecutive days.	Increased ROS s in the blood, liver and brain of mice as measured by DCFH-DA.<br>Increased GSH, TBARS and GST in blood and brain; TBARS in liver; GPx in blood,<br>Decreased SOD and catalase activity in blood, liver, and brain; GPx in liver and brain;GST in liver.

Setyawati et al., 2018 [223]	CellROX® Orange	*In vitro* exposure. The study intended to show that nano- TiO_2 can induce the Epithelial-to-mesenchymal transition (EMT) process in colorectal cancer cells (SW480).	Increased ROS in colo-rectal cancer cells in a dose-dependent manner. To further confirm the involvement of nano- TiO_2-induced ROS production in TGF-β pathway activation, cells were treated with N-acetyl cysteine (NAC), an ROS scavenger and ≈3.6 fold reduction of the nano- TiO_2-induced ROS production was observed after co-treatment of NAC and nano- TiO_2 (250, 3 days exposure). Concomitant with the drastic reduction in ROS level, NAC treatment significantly curbed the expression of P-ERK and P-p38 when compared to the groups receiving no NAC treatment. This supported the hypothesis that ROS upregulation derived from nano- TiO_2 exposure, drove the progression of the TGF-β-mediated EMT process in these colonic epithelium cells.
Shrestha et al., 2016 [120]	DCFH-DA	Ex- vivo oral exposure. In this study, four kinds of TiO2 nanorods (TiO2 NRs), with similar aspect ratios but different surface functional groups, i.e. amines (–NH2), carboxyl groups (–COOH) and poly(ethylene glycol) (–PEG), were used to study their interaction with rat bone marrow stem cells (MSCs) isolated from the bone marrow of Sprague-Dawley rats (6–8 weeks old) and exposed in vitro.	Iincreased ROS after exposure to TiO_2 nanorods
Hu et al, 2019 [74]	In vitro:<br>H2DCFDA;<br>total superoxide dismutase (T-SOD); glutathione synthetase (GSH);<br>methane dicarboxylic aldehyde (MDA)	This study explored the relationship between TiO_2 NP-induced ER stress and ROS generation both in vitro (murine cell line NIH/3T3 cells) and in vivo following oral exposure to TiO_2 (50 mg/kg b.w.) up to 26 weeks. Aimed also to assess TiO2 NP effect on plasma glucose levels.	Increased endoplasmic reticulum (ER) stress and ROS,<br>Decreased T-SOD levels and GSH levels in serum and liver;<br>Increased MDA serum and liver Oral administration of TiO_2 NPs initially induces endoplasmic reticulum (ER) stress followed by ROS generation, and finally,

	in vivo (serum and liver):<br>total superoxide dismutase (T-SOD); glutathione synthetase (GSH);<br>methane dicarboxylic aldehyde (MDA)<br>ROS related genes		IR and elevation of plasma glucose, signifying that ER stress is not induced by ROS. Treatment with 4-PBA inhibited TiO_2 NP-induced ER stress as well as ROS production and plasma glucose levels in mice in vivo and mouse NIH/3T3 cells in vitro.
Proquin et al. 2018 [8]	Gene expression analysis (distal colon)	*In vivo* oral exposure. This study aimed to investigate the molecular mechanisms after TiO_2 NP E171 exposure of BALB/c mice by gavage to 5 mg/kgbw/day for 2, 7, 14, and 21 days to 5 mg/kg bw/day.	altered gene expression . in oxidative stress related genes with upregulation of DEG such as Trp53, Cabin1 and Mapkapk3, by day 7; Ucp2 and Ucp3 were upregulated at 14 and 21 days and are activated when oxidative stress occurs.
Hu et al, 2018 [110]	gene expression of ROS-related genes (SOD1, SOD2, GSS, GCLC, GCLM);<br>Total Sod (T-SOD); GSH and MDA in sera and liver	*In vivo* oral exposure. TiO2 NPs impact on genome-wide pathways, especially the plasma glucose homeostasis-related pathways in mice orally administered with 10-200mg/kg b.w. of TiO_2.	Increase MDA and decreased of total SOD (T-SOD) and GSH in the serum and livers at dose 50 mg/kg b.w. and higher doses; The main genes enriched in ROS generation related GO terms and KEGG pathways were P450s (in the ER), suggesting that ROS is closely related to ER stress observed. 50 mg/kg b.w. and higher doses TiO2 NPs increased plasma glucose in mice; Similar expression levels of superoxide dismutase 1 (SOD1), SOD2, glutathione synthetase (GSS), glutamate cysteine ligase catalytic subunit (GCLC), and glutamate cysteine ligase modifier (GCLM) in all groups of animals;
El Dine et al., 2018 [200]	MDA<br>Reduced glutathione (GSH) (plasma)	*In vivo* oral exposure. Epigenetic changes of the genome, in the form of DNA methylation in liver tissue after oral administration of 100 mg/kg bw TiO_2 NPs (mixed rutile and anatase) in adult male albino rats, for 6 weeks.	TiO_2 NPs cause global DNA hypo-methylation in the liver. Increased MDA and reduced glutathione (GSH) in plasma.<br>Significant negative correlation between DNA methylation in liver tissue samples and GSH level.

			Significant positive correlation between DNA methylation in liver and level of MDA.
Shukla et al, 2014 [76]	Measurement of GSH<br>MDA<br>ROS by DCF-DA	*In vivo* oral exposure. Cyto and genotoxicity of anatase TiO_2 NPs at low doses (0, 10, 50 and 100 mg/kg) orally administered in mice for 14 days. Investigated biochemical parameters, oxidative stress, histopathological examinations, and the expression profile of different apoptotic /anti apoptotic proteins.	Increase MDA and ROS (increased at 50 and 100 mg/kg b.wt.) and decrease of GSH (decreased at 100 mg/kg b.wt).
Khorsandi et al, 2015 [223]	MDA, by thiobarbituric acid reactive substances (TBARS)<br>Superoxide dismutase (SOD) activity; Glutathione peroxidase (GPx) activity (liver)	*In vivo* oral exposure. This study investigated the hepatoprotective effect of glycyrrhizic acid (GA) against hepatic injury induced by titanium dioxide nanoparticles ($NTiO_2$) in rats orally administered with 300 mg/kg of $NTiO_2$ for 14 days.	Increased MDA and decreased SOD and GPx enzymes. Pretreatment of GA significantly ameliorated oxidative stress in hepatic tissue, and increased the activities of SOD and GPx.
Cao et al., 2019 [225]	CellROX®	*In vitro* exposure. Investigated the combined effect of TiO_2 (E171) and SiO2 (E551) and other toxic compounds (pesticide Boscalid) using a fasting food model (FFM), digested using a three phase GIT simulator. Digesta applied to in vitro tri-culture cellular models for toxicity, cellular uptake, translocation and ROS generation assessment of the pesticide boscalid alone or combined with TiO_2 (E171) or SiO2 (E551).	Compared with digesta from the FFM control (phosphate buffer alone), digesta from the FFM containing TiO_2 alone and boscalid alone caused a slight 1.20 ($p < 0.05$) and 1.08 ($p > 0.05$) fold increase in ROS production, respectively. The combinations of TiO_2 and boscalid at 10 and 150 ppm resulted in a 1.26 ($p < 0.01$) and 1.28 ($p < 0.01$) fold increase, respectively.
Ansari et al., 2017 [226]	Glutathione peroxidase (GPx);<br>Superoxide dismutase (SOD)<br>Catalase activity (Kidney)	*In vivo* oral exposure. Chemical and green synthesized TiO2 evaluated for its cytotoxicity and oxidative stress in LLC PK1 (Pig Kidney Epithelia) cells and Wistar rats.	In vivo exposure to synthesized TiO_2 led to oxidative stress in liver mice. TiNPs prepared by green route may exhibit additional biological effects by reducing oxidative stress and by limiting the elevated nephrotoxic markers.

Canli et at, 2017 [227]	Activity of CAT<br>Activity of SOD<br>Activity of GPX<br>Total, oxidized and reduced GSH levels (erythrocytes)	*In vivo* exposure. Effect of exposure to Al2O3, CuO and TiO_2 NPs on 14 serum biomarkers and 4 antioxidant enzyme activities in the erythrocyte of female rats, exposed via oral gavage (0, 0.5, 5, 50 mg/kg b.w./day) for 14 days.	Decreased activities of catalase and superoxide dismutase and increased glutathione peroxidase activity. No alterations in glutathione S-transferase activity
Tay et al., 2014 [50]	DCFH-DA	*In vitro* exposure. Cellular internalization, ROS generation, inflammatory response, and cytotoxic effects of nano- TiO_2 and nano-HA in n vitro TR146 human oral buccal epithelial cells.	Increased ROS at the higher concentrations (250 to 1250 µM).Stimulation of mitochondrial superoxide production.
Grissa et al., 2017 [141]	MDA;<br>Total antioxidant status (TAS);<br>Superoxide dismutase (SOD);<br>catalase (CAT) activity(plasma)	*In vivo* exposure. Efficacy of rosemary extract in ameliorating toxic effects induced on blood of TiO_2NP-intoxicated rats, via intragastric administration of rosemary extract 1 h before the intragastric administration of 100 mg/kg/day TiO_2 (10 nm) for 60 days.	Significant oxidative stress (reduced SOD and CAT and TAS, increased MDA)
Farcal, 2015 [32]	H2DCFDA	*In vitro* exposure. Six oxide NMs were tested in different laboratories using 12 cellular models representing 6 different target organs/systems (immune system, respiratory system, gastrointestinal system, reproductive organs, kidney and embryonic tissues), applying 10 different assays (cytotoxicity, embryotoxicity, epithelial integrity, cytokine secretion and oxidative stress.	No ROS generation in murine kidney (NRK-52E) or embryonic cells (NIH/3T3).
Azim et al, 2015 [78]	Malondialdehyde (MDA);<br>Glutathione (GSH) levels<br>(liver)	*In vivo* oral exposure. Nano-anatase TiO_2 (21 nm) was administered (150 mg/kg/day) for 2 weeks followed by the antioxidants idebenone, camosine and vitamin E alone or in combination for 1 month.	Increased MDA levels, and suppressed hepatic GSH level; inflammatory response via the activation of macrophages and the enhancement of tumor necrosis factor-and interleukin-6 levels.

Botelho, et al., 2014 [178]	GSHt, GSH and GSSG levels	*In vitro* exposure. DNA damage by Comet assay, cytotoxicity and oxidative stress after exposure to TiO_2 nanoparticle AGS human gastric epithelial cells, for 3h.	AGS cells show an increase in oxidized GSSG in 150 µg/mL of TiO_2 nanoparticles.
Jensen et al.,2018 [102]	MDA<br>BH4 tetrahydrobiopterin<br>Vitamin C (µM) (ascorbate + DHA)<br>(plasma)	*In vivo* oral exposure. Lean Zucker female rats were exposed once a week, 10 weeks to low-doses of E171(50 mg/kg bw/week) or high-dose E171 (500 mg/kg bw/week) by oral gavage.	No effect of markers of oxidative stress and vascular function on plasma.
Gandamalla et al., 2019 [82]	H2DCFDA	*In vitro* exposure. Human lung (A549) and colon (Caco-2) cells were exposed to test particles, TNP 18nm (TNP 18), TNP 30nm (TNP 30), and TNP 87nm (TNP 87) with a dose range 0.1–100 mg/ml and the effect of exposure was determined using MTT, LDH, and DCFH-DA methods.	Increased of ROS levels at higher doses 60 ($p<0.01$) and 100 ($p<0.01$) µg/ml compared to control for 4 h post exposure. Caco-2 cells produced a significant Increased ROS generation starting from dose 10 µg/ml for TNP 18. Increased ROS release significantly against the doses 30, 60, and 100 µg/ml for TNP 30 and TNP 87.<br>Relative release of ROS in Caco-2 cells observed in a dose and a size-dependent manner (TNP 18> TNP 30> TNP 87).
Jalili, et al., 2018 [86]	Reduced glutathione (GSH);<br>CellROX	*In vitro* exposure. Cytotoxic and genotoxic effects of two rutile TiO_2 NMs, differing in surface coating, NM103 (hydrophobic) and NM104 (hydrophilic), on intestinal (differentiated CACO-2) and hepatic cell (differentiated HEPARG) models, following 3 or 24 h treatments with concentrations of TiO2 NMs from 1.2 to 80 µg/cm^2.	No ROS generation or oxidative stress observed.
Hu et al., 2015 [142]	Total SOD (T-SOD);<br>GSH and MDA. (Serum and liver)<br>Phosphorylation of IRS1, Akt, JNK1, and p38 MAPK, analyzed by western blotting	*In vivo* oral exposure. Endocrine effects of oral administration to mice of anatase TiO_2 nanoparticles (0, 64 and 320mg/kg b.w./day to control low-dose and high-dose groups, respectively, 7 days per week for 14 weeks.	Increased ROS levels in serum and liver, as evidenced by reduced total SOD activity and GSH level and increased MDA content. The pathway by which TiO2 nanoparticles increase ROS-induced IR were included in the inflammatory response and

			phosphokinase, as shown by increased serum levels of TNF-α and IL-6 and increased phosphorylation of JNK1 and p38 MAPK in liver.
Filippi et al., 2015 [228]	DHE (Dihydroethidium) Assay; ESR spectroscopy	*In vitro* exposure. The impact of rutile TiO_2 (30nm) on liver C3A cells intermediary metabolism assessed by measurement of glucose production and glycolysis from endogenous glycogen and gluconeogenesis and glycolysis from lactate and pyruvate, Mitochondrial membrane potential; phosphoenolpyruvate carboxykinase (PEPCK) mRNA expression and intracellular reactive oxygen species (ROS) concentration.	4h exposure (dose range 0;0.50;2.50;10.0 μg/cm2) had no effect on hepatocyte intermediary metabolism and mitochondrial function, despite an inherent ability to generate ROS in a cell free environment as measured by EPR. No intracellular ROS production was observed in cells exposed to TiO_2.
Teubl et al., 2015 [124]	DHE (Dihydroethidium) Assay - Reactive Oxygen Species (superoxide and hydrogen peroxide), under light exclusion	*In vitro* exposure. Interactions of three TiO_2 NMs (NM 100, NM 101 and NM 105) with oral tissues were investigated. Physicochemical properties, intracellular localization and potential toxic effects were evaluated in in vitro buccal epithelial TR146.	Increased ROS in TR146 oral cells after exposure to NM 101 (7nm) and NM 105 (22nm), but not NM 100.
Zhang et al., 2010 [229]	Antioxidase glutathione peroxide (GSH-Px);<br>superoxide dismutase (SOD);<br>malonaldehyde (MDA);<br>DCFH-DA<br>(Serum, Liver,kidney,cortex,hippocampus)	*In vivo* oral exposure. The potential acute toxicity of the interaction between nanoparticle TiO_2 (50 and 120 nm) and lead acetate (PbAC) was investigated in adult mice following 7 days exposure to 5 g/kg body weight, by oral gavage.	Increased ROS in the liver but not in the kidney, cortex and hippocampus after exposure to the mixture of TiO_2 and PbAC. No increase of MDA levels.<br>No reductions of SOD and GSH-Px activity in liver and kidney but in the cortex and hippocampus. For both Tio2 alone, increased ROS was observed in all tissues, higher in liver and kidney and decreased GSH-Px and SOD was observed in serum and all tissues. TiO2-50 treatment significantly increased MDA level in the liver, kidney and hippocampus tissue compared with the control, and TiO2-120

			significantly increased MDA levels in kidney and hippocampus.
Abbasi-Oshaghi et al, 2019 [72]	*In vitro*:<br>DCFH-DA<br>GSH levels<br>In vivo (liver and intestine):<br>superoxide dismutase (SOD);<br>glutathione peroxidase (GPx);<br>catalase (CAT);<br>GSH levels;<br>Total oxidant status (TOS);<br>total antioxidant capacity (TAC);<br>MDA;<br>gene expression	The effects of TiO_2 NPs (rutile, 30 nm) were assessed *in vivo* on liver and intestine of normal male wistar rats following exposure to 10, 50 and 100 mg/kg, for 30 days, and in vitro in Caco-2 and HepG2 cells (0, 20,40,80,100, 150, 200, and 300 µg/mL) for 24h.	In vivo, decreased SOD, CAT, and GPX activity as well as GSH and TAC levels in rat liver (all dose tested) and decreased SOD, CAT (50mg/kg and 100mkg/kg) and GPX (all doses) activity as well as GSH and TAC levels (all doses) in rat intestine, both in a dose dependent manner. Increased MDA and TOS concentrations (10, 50, and 100 mg/kg) in the intestine and liver. Gene expressions of SOD, GPX, and CAT significantly reduced in all exposed groups in a dose-dependent manner in intestine and liver.<br>*In vitro*, increased ROS generation in Caco-2 and HEPG2 cells after exposure to the highest doses (100–300 µg/mL); decreased GSH levels in both cells.
Morgan et al., 2017 [57]	MDA<br>GSH<br>CAT<br>(testicles)<br>gene expression	*In vivo* oral exposure. The study investigated the mechanisms of TiO_2-induced genotoxicity and evaluated the effectiveness of Tiron in modulating toxic effects in adult male albino rats exposed to TiO_2 at 100 mg/kg/day orally, for 8 weeks.	Increased oxidative stress (increased MDA and decreased GSH); upregulation of Testin gene.
Dorier, et al., 2017 [81]	DCFH-DA;<br>antioxidant enzymes gene expression by RT-qPCR (CAT, SOD1; SOD2; GSR)	*In vitro* exposure. Exposure to E171 and two different types of TiO_2-NPs, either acutely (6-48 h) or repeatedly (three times a week for 3 weeks, using two in vitro models: (i) a monoculture of differentiated Caco-2 cells and (ii) a coculture of Caco-2 with HT29-MTX mucus-secreting cells.	Increased ROS in differentiated Caco-2 cells, following acute exposure to A12, P25 (50 µg/mL) and E171 (all concentrations) for 48h, and to P25 for 24h, and following repeated exposure to E171 at all concentrations (dose dependent);<br>In CACO-2/HT29-MTX-E12 co-culture, acute exposure increased ROS for all time points (6h,24h and 48h) for P25 (50 µg/mL), and E171 (50 and 100

			µg/mL); and at 6 and 24h for A12 (50 µg/mL), and after repeated exposure to E171 at 50 and 100 µg/mL and to P25 at 50 µg/mL (dose dependent). Antioxidant enzyme gene expression (CAT, SOD1; SOD2; GSR) not altered in Caco-2 cells, but differences were observed in co-culture either following acute or repeated exposure. In repeatedly exposed Caco-2/HT29-MTX, CAT and GSR were downregulated upon exposure to 10 µg/mL E171, and GSR and SOD1 were downregulated in response to 50 µg/mL E171. Exposure to 10 µg/mL A12 caused CAT and SOD1 downregulation: P25 only induced downregulation of SOD1. increased OS after 48h exposure correlating with intracellular accumulation of TiO_2.
Natarajan et al., 2015 [119]	H2DCFDA;<br>Manganese Superoxide Dismutase (MnSOD) activity mitochondrial membrane potential (MMP).	in vitro Ex-vivo exposure. Primary rat hepatocytes were exposed to three different TiO_2 NPs (commercially available rutile, anatase and P25) and cells were evaluated for hepatocyte functions and mitochondrial dynamics, namely redox signaling mechanisms.	Increased ROS for after exposure to all NMs (anatase> mixture>rutle) and decreased MnSOD scavenger enzyme activity after exposure to p25 and anatase), after 72h. Loss of MMP activity, critical for proper oxidative phosphorylation function to occur, suggests mitochondrial damage in primary hepatocytes and that increased ROS is not due to adaptive response.

Richter et al., 2018 [39]	CellROX®	*In vitro* exposure. The interaction between TiO_2 NMs exposure, beneficial bacterial populations (Lactobacillus rhamnosus GG), and intestinal function was investigated in co-cultures of Caco-2 and HT29-MTX. To complement the in vitro model, experiments were performed with D. melanogaster which has a gut much like the human gut.	and HT29-MTX did no ROS generation after exposure to a single dose of 0,14µg/mL for 4h to co-cultures of Caco-2 .
Ali et al, 2019 [77]	Malondialdehyde (MDA)<br>Glutathione (GSH)<br>Catalase (CAT)<br>Nitric oxide (NO)<br>superoxide dismutase (SOD)<br>(Liver)	*In vivo* oral exposure. The toxicity of TiO_2 NMs (21 and 80 nm) was evaluated after five days oral administration to different doses (50, 250 and 500 mg/kg body weight) in mice, via measurement of oxidative stress markers, liver function indices, aspartate and alanine aminotransferases (AST and ALT), chromosomal aberrations and liver histopathological pattern.	Decreased GSH level and increased CAT, NO and MDA, positively correlated with the TiO_2 NPs dose and negatively correlated with the particle size.
Morgan et al., 2017 [151]	MDA;<br>Superoxide dismutase (SOD);<br>Glutathione peroxidase (GPX) activity;<br>reduced glutathione (GSH);<br>protein content<br>(kidney)	*In vivo* oral exposure. The effect of anatase TiO_2 NMs (10 nm) was evaluated after 60 days of oral administration of 100 mg/kg b.w\day to albino Rat. The investigation was performed to assess the efficacy of tiron against TiO_2 NPs induced nephrotoxicity.	Depletion of renal antioxidant enzymes; increased MDA concentration; up-regulation in fibrotic biomarkers TGFb1 and MMP9.
Talamini et al., 2019 [58]	superoxide anion production<br>(Liver, stomach, and the whole intestine)	*In vivo* oral exposure. This study investigated whether repeated oral administration of E171 to mice at a dose level (5 mg/kg body weight for 3 days/week for 3 weeks) comparable to estimated human dietary exposure, results in TiO_2 deposition in the digestive system and internal organs, and in	Increased superoxide production and inflammation were observed in the stomach and intestine, but not in the liver.

		molecular and cellular alterations associated with an inflammatory response.	
Abdelazim et al., 2015 [147]	Total antioxidant capacity (TAC); Glutathione-S-transferase (GST); Total Nitrite/Nitrate (NOx) (liver)	In vivo oral exposure. The study investigated the effects of individual and combined doses of idebenone, carnosine and vitamin E on ameliorating biochemical indices of TiO2 NPs in mice liver, after oral exposure to 150 mg/ Kg/day for 14 days.	TiO2 induced oxidative stress: reduced GST in the liver; increasedTAC and NOx levels.
Winter et al., 2010 [106]	DCFH-DA	*In vitro* exposure. in vitro effects of micron-sized and nano-sized TiO_2 particles exposure (5, 20 and 40 mg/cm^2) to intestinal dendritic cells (DC) derived from bone marrow of C57BL/6 mice.	Increased ROS in intestinal DC after exposure to 20 and 40 mg/cm2 for 18h.
Wang et al., 2011 [97]	DCFH-DA	*In vitro* exposure. The toxicity of nano-TiO_2 was investigated using a primary culture of channel catfish hepatocytes and human HepG2 cells, using phase contrast inverted microscopy, MTT assays, ROS assays, and flow cytometric assays.	Increased intracellular ROS following exposure for 24 and 48 h. After 24 h, only dose 50ug/ml was increased.
Hu et al, 2020 [148]	total SOD (T-SOD), GSH, and MDA. (serum and liver	In vivo oral exposure. This study explored the impact of age on toxicological effects of TiO_2 NPs, plasma glucose levels and the mechanism of effects TiO_2 NPs on plasma glucose, by comparing adult mice (age 10 weeks) and at developing stage (age 3 weeks) after treatment with 50 mg/kg bw TiO_2 NPs.	Increased ROS earlier in developing mice than in adult mice. By week 8, the T-SOD in the serum and the GSH in the liver of the younger mice decreased significantly while MDA increased significantly. At week 26, the significant reductions in T-SOD and GSH and increased MDA content occurred in both mice groups. TiO2 NPs increased ROS linked with activated NFkB, JNK and p38 MAPK pathways.

Kim et al., 2019 [121]	DCFH-DA	*In vitro* exposure. The molecular mechanisms underlying the pro-inflammatory effect of TiO_2 -NP exposure and its capability to induce cyclooxygenase-2 (COX-2) was investigated in periodontal ligaments cells (PDL).	Dose dependentIncreasedintracellular ROS. ROS, concomitantly overproduced by TiO_2-NPs, induce COX-2 expression through activation of NF-κB signaling, which may contribute to the inflammatory effect of PDL cells.
Gu et al., 2015 [230]	total superoxide dismutase (T-SOD);<br>glutathione (GSH);<br>methane dicarboxylic aldehyde (MDA).<br>(liver and serum)	*In vivo* oral exposure. in vivo study with CD-1 mice orally administered with 64 mg/kg bw/day of TiO_2 FPs greater than 100 nm in size and anatase TiO_2 NMs (18nm), for 28 weeks, to address their effects on plasma glucose levels and ROS levels in liver and serum.	Decreased T-SOD and glutathione (GSH); increased MDA.
Martins et al., 2017 [59]	Glutathione peroxidase (GPx) activity<br>Glutathione (GSH) assay<br>Catalase (CAT) activity<br>(blood)	*In vivo* oral exposure. The redox parameters and genotoxic effects (comet assay) in male Wistar rats treated with either AgNP or TiO_2 NP (0.5 mg/kg/day) individually, as well as under a co-exposure scenario, for 45 days were evaluated. Tissue biodistribution of Ag and Ti was measured.	No effects on the redox status.
Hu et al., 2016 [60]	total superoxide dismutase (T-SOD); glutathione (GSH) and methane dicarboxylic aldehyde (MDA).<br>mRNA: (SOD1, SOD2, GSH S-transferase (GSS) (plasma)	*In vivo* oral exposure. This study explored the effect of TiO_2 NPs in plasma glucose levels via ROS, after oral administration of 64 mg/kg bw/day of TiO_2 NPs for 18 weeks.	Decreased T-SOD and increased MDA from 10 week onwards in plasma, with concomitantly increasing in plasma glucose. Possibly ROS activated inflammatory cytokines and phosphokinases, and thus induced insulin resistance, resulting in an increase in plasma glucose. No effect in the mRNA expressions of ROS-related markers.
Jia et al., 2017 [98]	superoxide anion (O2–) levels, catalase activity (CAT), malondialdehyde (MDA); H_20_2<br>mRNA levels of SOD, CAT, GSHPx and GST (Liver)	*In vivo* oral exposure. This study evaluated the liver, brain, and embryo toxicity and the underlying mechanism of nano- TiO_2 after exposure to 5, 10, 50 mg/kg)/daily for 60 days.	Increased O2−, H_2O_2, NO and MDA and decreased mRNA levels of SOD, CAT, GST and GSHPx genes in the livers of mice at the higher doses (10 or 50 mg/kg).

Pereira et al, 2018 [95]	H2DCFDA (ROS; RNS production/accumulation in Mitochondria<br>GSH/GSSG ratio<br>(liver)	*In vivo* oral exposure. This study investigated the effect of AgNPs and Ti NPs exposure with the mitochondrial respiration chain and how this interaction interferes in the bioenergetics and oxidative state of the organelles after subchronic exposure. Mitochondria were exposed to 100 µg/kg/day NPs by gavage treatment for 21 days.	No effect in ROS generation using H2DCFDA; decreased GSH/GSSG ratio. Extended co-exposure of mitochondria to the NPs maintained increased ROS levels and depleted the endogenous antioxidant system. The AgNPs and TiNPs acted synergistically—the intensity of the toxic effect on the mitochondrial redox state was more significant in the presence of both types of NPs.
Moradi et al, 2019 [143]	Total antioxidant capacity (TAC); total oxidant status (TOS); Malondialdehyde (MDA); Catalase activity; superoxide dismutase (SOD) activity; glutathione peroxidase (GPx) activity (liver)	*In vivo* oral exposure. This study investigated the hepatoprotective effects of vitamin E and vitamin A against hepatotoxicity induced by nano TiO_2 in Male Wistar rats after exposure to 300 mg/kg of TiO_2 for two weeks by gavage.	In liver, hepatic injury, increased TOS and MDA, decreased TAC and antioxidant enzymes (CAT, SOD GPx); Elevation of inflammatory mediators.
Chalew et al., 2013 [84]	DCFH-DA	*In vitro* exposure. Cell death, oxidative stress, and inflammatory effects of silver (Ag), titanium dioxide (TiO_2), and zinc oxide (ZnO) NPs was investigated on human intestinal Caco-2 and SW480 cells after 4h and 24h exposure to 0-100 µg/mL.	In Caco-2 and SW480 cells no ROS generation was observed after exposure to 0-100 µg/mL P25 TiO_2 (80% rutile/20% anatase; 21 nm) for 4h and 24h.
De Angelis et al., 2012 [80]	CM-H2DCFDA	*In vitro* exposure. The cytotoxicity and ROS formation induced by zinc oxide (ZnO) and titanium dioxide (TiO_2) NPs on Caco-2 cells was evaluated, along with cellular uptake, after 6 and 24h exposure to 1-20 µg/cm2 (6.4–128.0 µg/mL).	Increased ROS after 6 h treatment (dose dependent), but not after 24h treatment.
Shahin et al, 2017 [114]	GSH;<br>MDA (by the thiobarbituric acid assay)<br>(prostate and testicles)	*In vivo* oral exposure. This study investigated the effect of short-term oral exposure to nano TiO_2 on Wistar rat prostate and testis, and the associating reproductive-related alterations after exposure to 50 mg/kg/day for 1, 2 and 3 weeks. The study also	Prostatic and testicular significant time dependent increase in MDA content in the 1, 2 and 3-week groups (prostatic) and in the 2 and 3-week groups (testicular), relative to the corresponding control. Time dependent decreased Prostatic GSH level by

		evaluated the potential ameliorative effect of the natural flavonoid, morin, on n TiO_2-induced aberrations.	the 1, 2 and 3 week regimens showing 14.3%, 35.4% and 47.1% reduction, respectively, compared with the control. For testicular GSH content, rats exhibited differential response to the different tested regimens with significantly decreased testicular GSH in week 1 with significant 15.3% and 93% elevations in testicular GSH levels in week 2 and 3, respectively.
Setyawati et al., 2015 [108]	DCFH-DA	*In vitro* exposure. The effects of nanosized food-additives— SiO_2, TiO_2, and ZnO— were investigated on the intestinal systems (DLD-1, SW480, and NCM 460 cells) using cell viability and oxidative stress assays, gene-expression profiling, cell cycle analysis and protein expression.	Dose dependent Increase of ROS (250 and 1000 µM) in three intestinal cell models (DLD-1, SW480 and NCM 460, for the latter after 1000 µM).
Al-Rasheed et al., 2013 [145]	Reduced glutathione (GSH)content and Nitric Oxide (NO) (liver and serum)	*In vivo* oral exposure. Toxic impacts of TiO_2-NPs on rat kidneys and the possible prophylactic role of either quercetin or idebenone, after TiO_2-NPs administered orally (600 mg or 1 g/kg body weight) for 5 consecutive days.	Renal injury related to TiO_2-NPs toxicity stimulates the production of NO in serum of TiO_2-NPs intoxicated rats versus normal ones. The low and high doses of TiO_2-NPs caused GSH depletion in renal tissue.
Murugadoss, et al., 2020 [75]	GSH depletion in vitro and in vivo.	Tested two anatase TiO_2 NPs with different primary sizes (17 and 117 nm) and prepared ad-hoc suspensions composed of small or large agglomerates with similar dispersion medium composition. For in vitro testing, human bronchial epithelial (HBE), colon epithelial (Caco2) and monocytic (THP-1) cell lines were exposed to these suspensions for 24h; In vivo, C57BL/6JRj mice were exposed via oropharyngeal aspiration or oral gavage to TiO_2 suspensions for 3 days.	*In vitro*: large agglomerates of TiO_2 induced stronger responses than small agglomerates for glutathione depletion, IL-8 and IL-1Œ≤ increase, and DNA damage in THP-1, while no effect of agglomeration was observed with 117nm TiO_2.<br>*In vivo*: No effect on the GSH levels observed in liver and lung of mice.

Morgan et al., 2018 [231]	MDA, superoxide dismutase (SOD) activity, reduced glutathione (GSH); glutathione peroxidase enzyme (GPx) activity (liver)	In vivo oral exposure. This study investigated the Tiron modulatory role against apoptotic damage induced by TiO_2 NPs in rat livers, after exposure to 100 mg/kg/day of TiO_2 NPs for 60 days.	Increased oxidative stress through increasing MDA and decreasing GSH andSOD and GPx enzymes, in the liver. Upregulation of the proapoptotic Bax gene; and downregulation of the antiapoptotic Bcl-2 gene.
Chen et al., 2019 [94]	Oxidative stress biomarkers in the liver tissue GSH; GSSG;GSH/GSSG; GSH-Px; MDA; SOD;	*In vivo* oral exposure. This study aimed to illustrate the hepatotoxicity induced by TiO_2 NPs and the underlying mechanisms in rats administered daily with 0, 2, 10, 50 mg/kg daily ofTiO_2 NPs (29 nm), for 90 days. Changes in the gut microbiota and hepatic metabolomics were analyzed to explore the role of the gut-liver axis in the hepatotoxicity induced by TiO2 NPs.	Oxidative stress state induced by subchronic oral exposure to 10 and 50 mg/kg BW TiO2 NMs: Decreased GSH and GSH/GSSG at 10 and 50 mg/kg bw doses with increased GSSG; Increase GSH-Px at 10 and 50 mg/kg bw dose and SOD at 10 mg/kg bw dose; increased of MDA at 50 mg/kg bw dose.
