# Supplementary material for: Adverse Outcome Pathways Associated with the Ingestion of Titanium Dioxide Nanoparticles—A Systematic Review
Source: Nanomaterials (Basel). 2022 Sep 21;12(19):3275. doi: 10.3390/nano12193275 (PMC9565478; doi:10.3390/nano12193275)
Supplement: Supplementary file 1 [file nanomaterials-12-03275-s001.zip › Table S4.pdf]

Table S4– Studies reporting the cytotoxicity/apoptosis and cell death of TiO2-NPs in intestinal context.

Reference	Study description	Main findings concerning cytotoxicity/apoptosis/cell death
Proquin et al. 2017 [85]	Nano-sized and micro-sized fractions of E171 were tested for their contribution in ROS formation and DNA damage in an in vitro model using human Caco-2 and HCT116. The cytotoxicity of TiO2 to Caco-2 cells was determined using three different tests: lactate dehydrogenase (LDH), thiazolyl blue tetrazolium bromide (MTT) and Trypan blue assay, while in HCT116 cells was determined by Trypan blue assay.	E171 was cytotoxic in Caco-2 cells at a lower concentration of 14.3 µg/cm2 and presented no toxic effects in HCT116 cell cultures at concentrations of 5, 10, 50 and 100 µg/cm2
Gerloff et al. 2012 [87]	The potential of five TiO2-samples to induce cytotoxicity, oxidative stress, and DNA damage was evaluated in human intestinal Caco-2 cells. Cytotoxicity was determined by the lactate dehydrogenase (LDH) assay as well as the water-soluble tetrazolium salt (WST-1) assay	In the LDH assay, only the two anatase/rutile containing samples induced low but significant membrane damage at 4 or 24h time points but high concentrations only. In the WST-1 assay, after 24h all samples, except for the fine powder, were found to reduce metabolic activity. At equal surface area, in both cytotoxicity assays the anatase/rutile containing samples were found to be more toxic to the cells than the pure anatase samples.
Song et al. 2015 [44]	The cytotoxicity and translocation of two native TiO2 NPs, and these two TiO2 NPs pretreated with the digestion simulation fluid or bovine serum albumin were investigated in undifferentiated Caco-2 cells, differentiated Caco-2 cells and Caco-2 monolayer. The cell viability was evaluated by a WST-8 cell counting kit and the LDH test kit was used to assess the cell membrane integrity after 24h exposure.	The cytotoxicity of TiO2 NPs was low and was not concentration-dependent. The exposure to TiO2 NPs did not affect the function of the Caco-2 monolayer. The toxicity of TiO2 NPs to the monolayer is very similar to that of differentiated Caco-2 cells, inferring that differentiated Caco-2 cells are more suitable in vitro models for reflecting the cytotoxicity of NPs to the intestine.
Pedata et al. 2019 [37]	Conceivable mechanisms by which nano-sized TiO2 particles affect physiological function of the intestinal epithelium layer were analyzed using the differentiated Caco-2 cell line as a model of the human intestinal mucosa	Exposure to 42 µg/mL or 84 µg/mL of TiO2-NP produced a prompt effect at 4h and a substantial cytotoxicity at 24 h recognized by a reduction of ATP levels. The study confirmed that TiO2-NPs are preferentially entrapped by intestinal cells with detrimental effects on paracellular

	to identify the biological response triggered by TiO2 particles. Cytotoxicity was determined by using the Vialight Plus bioluminescence assay Kit in the presence or absence of 42 or 84 µg/mLTiO2-NPS for 4, 24, and 48 hours exposure.	permeability, cell viability and cytokines production, supporting the idea that nano-sized TiO2 is toxic to the human gastrointestinal tract.
MacNicoll et al. 2015 [47]	The study investigated the uptake and biodistribution of nano- and larger-sized titanium dioxide (TiO2) using an in vitro model of gut epithelium and in vivo in rat. The cytotoxicity was determined using the WST-1 assay on Caco 2 cells.	TiO2 nanoparticles are not likely to be absorbed in the GI tract following oral intake, and are likely to be excreted in the faeces over a period of time. TiO2 nanoparticles tested decreased the viability of Caco-2 cells with increasing nanoparticle concentration but these effects were most prominent for larger sizes.
Brown et al. 2019 [88]	The ToxTracker assay (mechanism-based reporter assay based on embryonic stem cells that uses GFP-tagged biomarkers for detection of DNA damage, oxidative stress and general cellular stress) was used to evaluate the potential hazardous of vegetable carbon (E153) and food-grade TiO2 (E171). Cytotoxicity was determined by the WST-1 assay in HepG2 and Caco-2 cells after 24h exposure.	The exposure of the stem cells to E171 for 24 h resulted in significant cytotoxicity at concentrations above 7.8 µg/cm2. The conventional toxicity assay verified very little cytotoxicity to either HepG2 or Caco-2 cells exposed to E171.
Manivannan et al. 2019 [177]	Swiss albino male mice were gavaged TiO2-NP at sub-acute concentration (0.2, 0.4 and 0.8 mg/kg body weight) over a period of 28 days and cytotoxicity and DNA damage (in multiple organs such as liver, spleen, thymus, afferent lymph node, and bone marrow cells), structural chromosomal aberration and clastogenic effects (bone marrow cells) were evaluated.	The mitotic index was affected upon exposure to concentrations (0.4–0.8 mg/kg body weight) of rutile TiO2-NP.
Tada-Oikawa et al. 2016 [83]	The effects of exposure to five TiO2-NPs of different crystal structures (anatase and rutile) and sizes (anatase, 50 and 100 nm and rutile, 50 nm) were determined in human epithelial colorectal adenocarcinoma (Caco-2) cells and THP-1 monocyte-derived macrophages. Cell viability was determined after incubation with the dispersed TiO2	The incubation of THP-1 macrophages with anatase TiO2 particles, of primary diameter of 100 nm, resulted in significantly reduced cell viability compared with rutile TiO2. The exposure of Caco-2 cells in the presence of anatase TiO2 with 50 nm, significantly reduced the cell viability after 72 h but no change in cell viability was evident following 24 h-exposure.

	particles at a concentration ranging from 1 to 50 µg/mL for 24 or 72 h, by MTS assay.	
Gerloff et al. 2009 [116]	The cytotoxic and DNA-damaging effects of several types of nanoparticles and fine particles relevant as food additives (as TiO2) as well as carbon black were investigated in human intestinal Caco-2 cells. The cytotoxicity was determined by using the LDH and the WST-1 assays following exposure of 4 and 24 h with 20 and 80 mg/cm2 particles.	After 24 h exposure to SiO2, TiO2 and ZnO induced a significant LDH release and thus loss of membrane integrity at both treatment concentrations. The results of WST-1 assay showed that all particles with the exception of MgO, reduced metabolic activity and thus induced toxicity. Both the LDH and WST-1 assays revealed a clear dose-dependent cytotoxic effect of TiO2-NPs after 24 h incubation.
Koeneman et al. 2010 [31]	Both toxicity and mechanistic studies were employed to investigate possible pathways by which titanium dioxide (TiO2) nanoparticles could cross the epithelium layer and whether the exposure of cells to TiO2 nanomaterials results in a rise of intracellular-free calcium. Fluorescent reporter dyes that could distinguish between live and dead cells were applied to test toxicity in Caco-2 cells.	Results of the live/dead assays indicated that Caco-2 cells remain alive after both acute (70h) and chronic (10 days) exposure to TiO2 (1, 10, 100, 1000 µg/mL).
Mohamed et al. 2015 [162]	Genotoxicity and possible induction of chronic gastritis by nano-TiO2 was investigated in mice orally administered 5, 50 or 500 mg/kg body weight for five consecutive days. Then mice from each dosage group were sacrificed 24 hours or one or two weeks after the last treatment.	Orally administrated nano-TiO2 in mice was retained in the stomach up to at least 2 weeks after stopping its administration, resulting in the development of chronic gastritis as evidenced by gradual dose- and time-dependent apoptotic DNA damage, histopathological changes, and oxidative stress induction in mice gastric cells. Dose- and time-dependent mutation induction in the p53 gene revealed that nano-TiO2 induced apoptosis is a p53 mediated process
Fujiwara et al. 2015 [109]	The toxicity of anatase and rutile TiO2-NPs was determined with and without concomitant UV irradiation. The production of oxidative stress and cytokines were investigated in vitro using CT26 and LL2 cells and the possible antitumor application of TiO2-NPs was examined in vivo in rat lungs and mouse colon.	The treatment of CT26 cells with or without UVB irradiation resulted in TiO2 concentration dependent cytotoxicity and this effect was enhanced by the UVB treatment. The cytotoxic effect of anatase TiO2-NPs was found to involve the induction of apoptosis in both LL2 and CT26 cells.

Orazizadeh et al. 2014 [79]	The hepatoprotective effect of glycyrrhizic acid (GA) against hepatic injury induced by TiO2-NPs was investigated in rats. TiO2-NPs-intoxicated rats received 300 mg/kg of TiO2-NPs for 14 days by gavage method.	GA by decreasing oxidative stress could effectively reduce necrosis and apoptosis of hepatocytes induced by TiO2-NPs.
Sohal et al. 2020 [34]	5 food-grade nanomaterials (TiO2, SiO2, ZnO, and two types of Fe2O3: rod-shaped and acicular) were administered to an *in vitro* mucus-secreting coculture (C2BBe1-Caco-2 subclone/HT29-MTX), to a triculture (C2BBe1/HT29-MTX/Raji B) and to a C2BBe1 (enterocyte) monoculture system to assess effects of exposure to each tested NM. Cellular Metabolic activity was assessed using the PrestoBlue® tetrazolium salt reduction assay and the cell membrane damage was determined by the LDH assay.	All NMs caused a significant reduction in cell metabolic activity which was reduced to a greater extent in C2BBe1/HT29-MTX co-cultures than in monocultures. In co-cultures exposed to TiO2, the metabolic activity was reduced to 78.2 ± 4.05 % of control. Increases in LDH release after TiO2 and SiO2 exposures were overall slightly more pronounced and significant in co-cultures than in monocultures.
Blevins et al. 2019 [139]	The potential of thymol to protect against the TiO2-NP-induced testicular damage was investigated in rats randomly divided into four groups: control group, TiO2 (100 mg/kg BW/day) group, TiO2 + thymol (10 mg/kg BW/day) group, and TiO2 + thymol (30 mg/kg BW/day) group that received orally TiO2-NP for 60 days.	Intragastric administration of TiO2 caused a significant decrease in sperm quality, widespread histopathological alteration, and significantly induced oxidative stress as manifested by elevated MDA levels and a remarkable decline in antioxidant enzyme activities such as CAT, SOD, and GPx, and also FRAP and GSH levels in testis tissue. Treatment with thymol was shown to be effective in preventing the toxic effects induced by TiO2-NPs.
Dorier et al. 2019 [201]	The *in vitro* impact of the food additive E171 and NM105 was investigated in a co-culture of Caco-2 and HT29-MTX cells. Cell viability was assessed by MTT assay with an exposure time of 6 or 48 h.	The overall toxicity of TiO2-NPs is low, even at high concentrations up to 200 µg/mL and does not induce any loss of cell viability.
Elbastawisy et al. 2014 [160]	Histomorphological alterations occurring in maternal and neonatal pulmonary distal airspaces were determined in Wistar rats after maternal administration of TiO2-NP.	After TiO2-NPs intake occurred thickening of the pulmonary interstitium, changes of the lamellar inclusion ultrastructure, pneumocyte apoptosis, and the presence of interstitial particle-laden macrophages. TiO2-induced alveolar epithelial cell apoptosis might be the cause of abnormal thinning of the alveolar septa.
Cao et al. 2020 [91]	The cytotoxicity and changes to the proteomic profile in an *in vitro* small intestinal epithelium model (co-culture of	At starting concentrations of 150 µg/mL and 300 µg/mL (final concentrations in the digesta-cell culture medium mixture) in FFM or SFM

	Caco-2 and HT29-MTX cells with Raji B cells) were investigated after exposure to digested food models containing TiO2 (E171) with an average size around 110 nm. Two different food models (fasting food model (FFM) and a standardized food model (SFM)) were evaluated, and a gastrointestinal tract (GIT) simulator was used to mimic oral, gastric, and small intestinal digestion. Cytotoxicity test was performed after 24 h treatment in transwell tri-cultures using the Pierce LDH assay kit.	of, TiO2 (E171) caused little or no cytotoxicity in the tri-culture small intestinal epithelium model. TiO2-NPs may have adsorbed some of the constituents in SFM, thereby masking harmful surface atoms and groups, and otherwise altering surface characteristics and possibly cellular uptake resulting in reduced cytotoxicity.
Chen et al. 2013 [103]	A method to separate TiO 2-NPs from gum was established, then obtained qualitative and quantitative characterization and cytotoxicity data of TiO2-NPs in gum. The human gastric epithelial cell GES-1 and the human epithelial colorectal adenocarcinoma cell Caco-2 were selected to evaluate the possible toxicity of TiO2-NPs of gum in the gastrointestinal tract. The toxicity was tested quantitatively by measuring the cell viability, lactate dehydrogenase (LDH) leakage from cells, and reactive oxygen species (ROS) level.	For GES-1 cells, TiO2-NPs of Gum-1 did not induce visible viability loss and membrane damage even at a concentration of 200 μg/mL. All TiO2 samples separated from gums did not induce any significant cell viability loss or membrane damage in both types of cells after 24 h of exposure.
Shrestha et al. 2016 [120]	Four kinds of TiO2 nanorods (TiO2 NRs), with a similar size and aspect ratio but different surface functional groups, i.e. amines (–NH2), carboxyl groups (–COOH) and poly (ethylene glycol) (–PEG), were used to study their interaction with rat bone marrow stem cells (MSCs). Cell viability was measured using the MTT assay for various concentrations of TiO2 NRs (5, 10, 20, 50 and 100 mg/mL) and exposure times of 24 h and 72 h.	The TiO2–core NRs showed noticeable cytotoxicity that increased along with the concentration and might be attributed to their high cellular loading amount. The toxicity was significantly lowered for the surface-modified TiO2 NRs, the cytotoxicity of TiO2–NH2 NRs was smaller than that of TiO2–core NRs at the same concentration and The TiO2–COOH and TiO2–PEG NRs showed a very limited influence on cell viability after 72 h incubation at all tested concentrations.
Evans et al. 2002 [133]	The cellular effects of TiO2, Ca2+, and lipopolysaccharide (LPS) alone, as well as the effects of the microparticle–toxin conjugate TiO2-Ca2+-LPS were examined using cultured intestinal lamina propria mononuclear (LPMNCs)	Isolated intestinal cells undergo rapid apoptosis and show a 2–3-fold increase in the caspase 1-induced release of IL-1β with physiological levels of luminal calcium which is further enhanced by the addition of bacterial LPS and the dietary microparticle TiO2.

	cells isolated from patients with and without inflammatory bowel disease (IBD).	
Hu et al. 2018 [110]	Whole transcriptomes of mice were tested after the oral administration of TiO2-NPs using RNA-seq and how the genome-wide pathways were affected, especially the plasma glucose homeostasis-related pathways. For the analysis of cell apoptosis, pancreatic and liver sections were stained with terminal deoxynucleotidyl transferase-mediated dUDP nick end-labeling (TUNEL).	TUNEL results showed that there were no apoptotic cells in mice pancreases and livers of any groups, that is, no cells showed a green color like the positive control.
Zhou et al. 2019 [232]	The investigation of follicular development, regulation of TGF-β-mediated signaling pathways, and expression of the pathway components was addressed by the exposure of female mice to different doses of nano TiO2 (1.25, 2.5, or 5 mg/kg body weight) via intragastric administration for 60 consecutive days.	Decreased p-mTOR, RPS6, p-RPS6, TSC1, and TSC2 protein expressions in response to nano TiO2 exposure, potentially underlies nanoparticle-induced inhibition of follicular development and oocyte apoptosis or follicular atresia in mice. Ovarian Foxo3a was upregulated, whereas the AKT level is suppressed by nano TiO2, which may promote oocyte apoptosis or follicular atresia and suppression of follicular growth in mouse ovary.
Abu-Dief et al. 2018 [233]	The biochemical, the histological and the histochemical changes in the liver after administration of different doses of TiO2 NPs were investigated and the possible protective role of Milk thistle (an herbal supplement used to treat liver and biliary disorders) against these effects was evaluated.	Mice treated with TiO2 showed signs of apoptosis and degeneration in the hepatocytes with nuclear changes and a significant increase in P53 and PCNA antibodies positive cells.
Shukla et al. 2014 [76]	The effects of TiO2-NPS from the oral exposure of mice (10, 50, 100 mg/kg b.wt.) for 14 consecutive days were analyzed for alteration in different hepatic enzymes, histopathological changes, oxidative stress, DNA damage, tumor suppressor and proapoptotic protein expression in liver cells.	It was observed a dose-dependent significant increase in the expression of proapoptotic proteins (Bax) and suppression of antiapoptotic proteins (Bcl-2). The modulation in the Bax:Bcl-2 ratio suggests that the primary apoptotic signal could be attributed to the increased p53 level. The observed dose-dependent significant increase in the level of initiator and effector caspase (caspase-9 and -3) resulted in a cascade of events that triggered cell death.
Watari et al. 2008 [135]	Both biochemical cell functional test and animal implantation test were performed to clarify the particle	Both biochemical cell functional test and animal implantation test showed the toxicity due to fine particles and its size dependence.

	size dependence of reaction of cells and tissue and micro/nanosizing effect of non-soluble materials such as Ti and TiO_2. Particles smaller (0.5, 3 µm) and larger (10, 50, 150 µm) than neutrophils were used to determine the relationship between cell and particle size with respect to cytotoxicity. Cell viability was measured by the LDH assay.	
Zhang et al. 2018 [133]	The effects of maternal exposure of TiO_2 NPs on the placentation were investigated. Mice were administered TiO_2 NPs by gavage at 0, 1 and 10 mg/kg/day from gestational day (GD) 1 to GD 13. Western blotting and TEM were used to assess the apoptosis of placenta.	TiO_2 NP treatment significantly inhibited the proliferation and induced apoptosis of placenta by the activation of Casp-3, upregulation of Bax and downregulation of Bcl-2 protein.
Cao et al. 2019 [225]	The effects of TiO_2 (E171) and SiO_2 (E551), on the cytotoxicity, cellular uptake and translocation of the pesticide boscalid were investigated. Fasting food model (phosphate buffer) containing the NM (1% w/w), boscalid (10 or 150 ppm), or both, was processed using a simulated *in vitro* oral-gastric-small intestinal digestion system which was applied to an *in vitro* tri-culture small intestinal epithelium model (Caco-2 and HT29-MTX cells with Raji B cells) to assess the effects on cell layer integrity, viability, cytotoxicity and production of reactive oxygen species (ROS).	At the low starting food concentrations of boscalid (10 and 150 ppm), TiO_2 and SiO_2 (1% w/w) none of these substances by themselves caused appreciable adverse effects in the small intestinal epithelial model. Cytotoxicity and ROS production in cells exposed to combined NM and boscalid were greater than in cells exposed to NMs or boscalid alone, and roughly equal to the sum of effects of individual treatments.
Ansari et al. 2017 [226]	The renal toxic effects of TiO_2-NPs prepared by chemical (sol gel technique) and green route were investigated in different experimental in vivo models viz., Wistar rats (100 mg/kg b.wt.; oral), LLC-PK1 cells (100 mg/mL) and isolated renal mitochondria (0.25, 0.5 and 1 mg/mL).	Toxicity was prominent in the mice treated with TiO_2-NPs prepared by conventional chemical route. TiO_2-NPs prepared by green route may exhibit additional biological effects by reducing oxidative stress and by limiting the elevated nephrotoxic markers.
Tay et al. 2014 [50]	The cellular internalization, inflammatory response and cytotoxic effects of TiO_2-NPs and nano-HA were characterized using TR146 human oral buccal epithelial	Cells treated with both NMs retained high cell viability >90% and percentage of necrotic cells did not fluctuate even at high NMs exposure. TiO_2-NPs treatment clearly induces a higher percent of early apoptotic cells in a dose dependent manner. TiO_2-NPs was observed to have a

	cells as an *in vitro* model. The percentage of apoptotic cells was determined by use of flow cytometry.	higher cytoplasmic localization and may trigger a defensive move by the cell to isolate these foreign entities until a critical threshold is reached, beyond which, the apoptotic events cannot be prevented anymore.
Tassinari et al. 2014 [117]	Possible reproductive and endocrine effects of short-term (5 days) oral exposure to anatase TiO2-NPs (0, 1, 2 mg/kg body weight per day) were explored in young, sexually mature rats.	The endocrine-active tissues are targets of TiO2 nanoparticles toxicity at oral dose levels relevant to human dietary intake. Such effects occurred in the absence of general toxicity, and with limited tissue deposition and damage in spleen, considered as indicators of TiO2 bioaccumulation.
Farcal et al. 2015 [32]	Six relevant NMs with different physico-chemical properties provided by the EC-JRC Nanomaterials Repository were tested in nine laboratories. The in vitro toxicity of NMs was evaluated in 12 cellular models representing 6 different target organs/systems (immune system, respiratory system, gastrointestinal system, reproductive organs, kidney and embryonic tissues). The toxicity assessment was conducted using 10 different assays for cytotoxicity, embryotoxicity, epithelial integrity, cytokine secretion and oxidative stress.	TiO2 (rutile) NMs showed minor effects to most of the cells studied, up to 72h of exposure. It was not possible to establish a consistent difference between the hazardous properties of the NM-103 and NM-104 in all the cell models adopted; a difference could be observed only in the case of alveolar macrophages (MH-S), with a slight increase of cytotoxicity for hydrophobic NM-104. Both NM-103 and NM-104 displayed some toxicity on the mouse embryonic stem cell line D3 (mES) and on mouse NIH/3T3 fibroblasts. A long term exposure (10 days) or repeated exposure (7 exposures within 21 days) of Caco-2 intestinal cells to both TiO2 NMs induced minor toxic effects, at least in the in vitro assays used.
Azim et al. 2015 [78]	The efficacy of idebenone, carnosine and vitamin E in ameliorating some of the biochemical indices induced in the liver of titanium dioxide nanoparticles (TiO2NPs) intoxicated mice was investigated. Nano-anatase TiO2 (21 nm) was administered (150 mg/kg/day) for 2 weeks followed by the aforementioned antioxidants either alone or in combination for 1 month.	The oral administration of TiO2-NPs trigger oxidative stress, inflammatory cascade, and potentially enhance the apoptotic machinery and DNA damage, resulting in severe liver injury. Idebenone, carnosine and vitamin E alleviated the hazards associated with TiO2NPs administration, with the combination regimen showing a relatively more potent effect.
Nejad et al. 2016 [118]	Effects of high intensity focused ultrasound (HIFU) combined with photocatalytic TiO_2-NPs were investigated in human oral squamous cell line HSC-2.	Cytotoxicity was enhanced in vitro by the combination of TiO2 and HIFU compared to each treatment alone. The cell toxicity had direct correlation with the concentration of TiO2 in the medium, as well as intensity and duration of HIFU exposures. In animal experiments, high intensity ultrasound induced TiO2 penetration into cytoplasm of the tumor cells.

Botelho et al. 2014 [178]	Proliferation, apoptosis, oxidative stress and genotoxicity of exposed AGS (gastric epithelial cancer) cells to TiO_2-NPs were assessed.	The exposure to TiO_2-NPs resulted in an increased proliferation of gastric epithelial cells. TiO_2 nanoparticles are likely to participate in a number of carcinogenesis-mediated processes, such as increased cell proliferation, decreased apoptosis, increased oxidative stress and increased genotoxicity, all of which are processes needed for cancer cell survival.
Gandamalla et al. 2019 [102]	Caco 2 and A549 cells were exposed to TiO_2 NPs (mixture of 80% anatase and 20% rutile) and 3 size (18, 30 and 87) with a dose range 0.1–100 µg/ml. The effect of exposure was determined using MTT, LDH, and DCFH-DA methods.	NPs induced a dose- and size-dependent cytotoxicity via decreased cell viability, increased LDH and ROS levels by in vitro methods.
Jalili, et al., 2018 [86]	Cytotoxic and genotoxic effects of two rutile TiO_2 NMs, differing in surface coating, NM103 (hydrophobic) and NM104 (hydrophilic), on intestinal and hepatic cell models, following 3 or 24 h treatments with concentrations of TiO_2 NMs from 1.2 to 80 µg/cm2	Although TEM demonstrated the presence of the two TiO_2 NMs within the cytoplasm, no significant cytotoxic or genotoxic were observed in either cell model.
Garcia-Contreras et al, 2014 [123]	Evaluation of the effect of TiO_2 NPs on the drug-sensitivity of oral squamous cell carcinoma and inflammation of human gingival fibroblasts (HGFs). Cytotoxicity was assessed by MTT.	TiO_2 NPs (0.05-3.2 mM) did not affect HGF cell viability, although TiO_2 NPs clusters were dose-dependently incorporated into the vacuoles of cells. NPs at concentrations higher than 0.2 mM exert an pro-inflammatory action against HGF cells, regardless of the presence or absence of IL-1β.
Teubl et al 2014 [124]	To study the interactions of 3 (TiO_2) particles (i.e. NM 100, NM 101 and NM 105) with oral tissues. Tested cells: Human buccal epithelial TR146. MTS and LDH assays were used.	Cell viability/integrity was not affected negatively (up to 200 µg/ml); however, NM 105 triggered the production of ROS independent of the concentration.
Susewind et al 2015 [115]	NM–101 TiO_2 nanoparticles (7–10 nm) were tested in a 3D co-culture of 3 human cell lines (Caco-2 and 2 human immune cells).<br>LDH and Resazurin assays were used.	NM101 TiO_2 nanoparticles did not induce toxic effects or Inflammation.

Abbasi-Oshaghi et al., 2019 [72]	This study evaluated the effects of titanium dioxide nanoparticles (TiO2-NPs) on the liver and intestine of normal rats. Cytotoxicity, ROS generation, and apoptosis rate were determined in HepG2 and Caco-2 cells.	TiO2-NPs in a dose-dependent manner increased cytotoxicity, oxidative stress, and apoptosis rate in Caco-2 and HepG2 cells. TiO2-NPs could have cytotoxic effects on the intestine and liver structure and function by inducing oxidative stress, inflammation, and apoptosis.
Bettini, et al., 2017 [3]	Rats orally exposed for one week to E171 at human relevant levels or NM 105 (anatase-rutile). To explain the growth-promoting effects on colonic preneoplastic lesions, authors tested whether E171 differentially affects the viability of normal or preneoplastic colon epithelial cells through the comparative cytotoxicity on non-mutated (Apc+/+) cells and genetically defined preneoplastic (Apc Min/+) cells using an MTT assay.	Titanium was detected in the immune cells of Peyer's patches (PP) as observed with the TiO2-NP model NM-105. Dendritic cell frequency increased in PP regardless of the TiO2 treatment, while regulatory T cells involved in dampening inflammatory responses decreased with E171 only, an effect still observed after 100 days of treatment. In all TiO2-treated rats, stimulation of immune cells isolated from PP showed a decrease in Thelper (Th)-1 IFN-gamma secretion, while splenic Th1/Th17. Exposure to food-grade TiO2 promotes colon carcinogenesis in vivo and induces preferential cytotoxicity in normal colon epithelial (Apc+/+) cells in vitro.
Morgan, et al., 2017 [57]	Adult male albino rats exposed to TiO2 at 100 mg/kg/day orally for 8 weeks	The results revealed high incidence of micronucleated RBCs, elevated oxidative stress parameters and up regulation of Testin gene.
Dorier, et al., 2017 [81]	Cells were exposed to E171 and 2 different types of TiO2-NPs, either acutely (6-48 h) or repeatedly (three times a week for 3 weeks, using two in vitro models: (i) a monoculture of differentiated Caco-2 cells and (ii) a coculture of Caco-2 with HT29-MTX mucus-secreting cells. Cytotoxicity was assessed using the WST-1 assay (0–200 µg/mL of particles for 6 h or 48 h); in the repeated exposure for monoculture trypan blue exclusion and co-culture (PI exclusion).	Results confirm that E171 damaged these cells, and that the main mechanism of toxicity was oxidation effects. Oxidative damage to DNA was detected in exposed cells, proving that E171 effectively induces oxidative stress; however, no endoplasmic reticulum stress was detected.
Giovanni et al., 2014 [105]	TiO2 Aeroxide P-25 NPs effect on human buccal epithelial cells (TR146) and human colon mucosal epithelial (NCM460). Cell viability evaluated by flow cytometry.	TiO2 NPs did not cause significant cytotoxicity toward the cells representative of the oral-gastrointestinal system.

Natarajan et al 2015 [119]	The cytotoxicity of three different TiO_2 nanoparticles (P25, anatase, and rutile) was evaluated in primary hepatocytes by the MTT assay.	The exposure of hepatocytes to TiO_2 nanoparticles showed a concentration and type dependent loss in viability in P25 treatment; 84% of cells were viable when exposed to 20 ppm concentration that decreased to 75% at 100 ppm concentration. Similarly in hepatocytes exposed to anatase nanoparticles, the cell viability decreased substantially from 85% in the 20 ppm concentration to 66% in 100 ppm. In rutile treatment, the loss in viability was concentration dependent but demonstrated the least severity, where the cell viability was approximately 80% in the 100 ppm treated samples.
Kaiser et al, 2013 [152]	The cytotoxic effects of nano-titanium dioxide were tested in gastrointestinal cells (CaCo-2) or immune system cells	When exposed to nano-titanium dioxide in concentrations up to 243 µg/mL for 48 h, the cells were not significantly affected.
Morgan et al 2017 [151]	The investigation was performed to assess the efficacy of tiron against TiO_2 NPs induced nephrotoxicity. Eighty adult male rats divided into four different groups were used: group I was the control, group II received TiO_2 NPs (100 mg\Kg BW), group III received TiO_2 NPs plus tiron (470mg\kg BW), and group IV received tiron alone.	The elevated renal indices detected in the study indicated the renal injury induced with TiO_2 NPs including renal apoptosis.
Abdelazim et al 2015 [147]	The study investigated the in vitro and in vivo effects of individual and combined doses of idebenone, carnosine and vitamin E on ameliorating some of the biochemical indices of nano-sized titanium dioxide (29 nm) in mice liver. The in vitro cytotoxic effect on hepatic cell lines (HepG 2) was investigated. Additionally, n-TiO_2 was orally administered (150 mg/kg/day) for 2 weeks, followed by a daily intragastric gavage of the aforementioned antioxidants for 1 month.	n-TiO_2 induced significant cytotoxicity in hepatic cell lines and elevated the levels of serum alanine aminotransferase (ALT), aspartate aminotransferase (AST), hepatic total antioxidant capacity (TAC) and nitrite/nitrate (NO_x) levels.
Winter et al 2011 [106]	To compare the in vitro effects micron-sized with nano-sized TiO_2 particles on intestinal dendritic cells.	Neither nanosized nor micronsized TiO_2 particles induced apoptosis in the cells.
Wang et al. 2011 [97]	The toxicity (cytotoxicity, apoptosis, ROS) of nano- ZnO, TiO_2, CuO and Co_3O_4 was investigated using a primary	The overall ranking of the toxicity of metal oxides to the test cells is as follows: $TiO_2 < Co_3O_4 < ZnO < CuO$.

	culture of channel catfish hepatocytes and human and human HepG2 cells.	
Chen et al. 2016 [131]	Various human oral and lung cell lines were selected to evaluate the cytotoxicity of treatment using different sizes and shapes of TiO2 NMs, including spheres (AFDC and AFDC300) and rods (M212 and cNRs).	Based on cell viability and endocytic behavior results, treatment with all the selected TiO2 NMs were nearly non-toxic to the oral cell lines. However, high cytotoxicity was observed in lung cells with M212 and AFDC treatments at 50 μgmL^{-1}
Kim et al 2019 [121]	To assess whether TiO2-NPs induce cyclooxygenase-2 (COX-2) and investigated the molecular mechanisms underlying the pro-inflammatory effect of TiO2-NPs on human periodontal ligament (PDL) cells.	Treatment of the cells with TiO2-NPs at relatively low concentration (10 μg/mL) for 24 hr did not affect the cell viability. However, incubation with high concentrations (20-50 μg/mL) of TiO2-NPs for 24 or 48 hr resulted in cytotoxic effects. ROS, concomitantly overproduced by TiO2-NPs, induce COX-2 expression through activation of NF-κB signaling, which may contribute to the inflammatory effect of PDL cells.
Bideskan et al [132]	Pregnant and lactating Wistar rats received intragastric TiO2-NPs (100 mg/kg body weight) daily from gestational day (GD) 2 to (GD) 21 and postnatal day (PD) 2 to (PD) 21 respectively. The effects of TiO2-NPs on hippocampal neurogenesis were evaluated including apoptosis.	Maternal exposure to TiO2-NPs significantly impacts hippocampal neurogenesis and apoptosis in the offspring.
Schneider et al 2017 [107]	investigated the toxicological behavior of metal nanoparticles (gold, silver) and metal oxide nanoparticles (copper oxide, zinc oxide, TiO2) in vitro in human colorectal adenocarcinoma cells (HT29).	TiO2-NPs showed no significant effect on cell viability and percentages of early and/or late apoptotic cells not higher than 20%.
Agnihothram, et al., 2016 [122]	Effect of anatase TiO2 NPs RAW 264.7 macrophages viability assessed by trypan blue exclusion. Dose range: 20 μg/ml and 2 μg/ml based on extrapolation of data indicative of the amount of human daily NP consumption.	Cell incubation with 20 μg/mL anatase caused a 30% reduction in cell viability; 2 μg/mL anatase did not alter morphology or viability. No genotoxic insults due to anatase TiO2.
Hu et al, 2016 [60]	The study aims to explore whether TiO2 NPs increase plasma glucose via ROS.	Oral administration of TiO2 NPs does not induce cell apoptosis in the pancreas and liver of mice.
Liang et al 2016 [126]	Three engineered metal oxide nanoparticles (NPs) with similar size but significantly different solubility were used to study the cellular uptake, toxicity and immune activation of Raw 264.7 cells.	In general, the TiO2-NPs did not have obvious cytotoxicity. However, they can induce strong immune responses of Raw264.7 cells, leading to higher expression of TNF- and IL-6, especially at relatively longer incubation time.

Chalew et al. 2013 [86]	Using NPs spiked into synthetic water and cell culture media, cell death, oxidative stress, and inflammatory effects of silver (Ag), titanium dioxide (TiO2), and zinc oxide (ZnO) NPs were investigated on human intestinal Caco-2 and SW480 cells.	TiO2 NPs were toxic only at 100 mg/L to both cell lines.
De Angelis et al, 2013 [80]	The toxicity (cell viability, uptake, ROS, inflammation) induced by zinc oxide (ZnO) and titanium dioxide (TiO2) NPs on Caco-2 cells.	TiO2 NPs were not cytotoxic and they induced a significant ROS production in Caco-2 cells only after 6 h.
Cai et al 2019 [111]	To determine the effects of the exposure route on the biodistribution, bio- persistence, and toxicology of nanoparticles (titanium dioxide, zinc oxide, and zirconium dioxide) in both mouse dams and their offspring. Oral and airway exposure routes were tested using gavage and intranasal administration, respectively.	All of the nanoparticle exposures induced high apoptosis rates for the mammary gland.
Shahin et al. 2017 [114]	The study investigated the effect of short-term oral exposure to nano-sized titanium dioxide (nTiO2) on Wistar rat prostate and testis, and the associated reproductive-related alterations.	Intragastric exposure to nTiO2 for 1, 2 and 3 weeks resulted in considerable alterations in both intrinsic and extrinsic apoptotic machineries, as manifested by alterations in prostatic and testicular gene expression levels of the anti-apoptotic factor, Bcl-2, the pro-apoptotic factor, Bax, the death receptor, Fas, and the key executioner of apoptosis, caspase-3.
Setyawati et al 2015 [108]	To investigate the effects of common nanosized food-additives—namely, SiO2, TiO2, and ZnO on human carcinoma epithelial cells (SW480 and DLD-1 and the normal human intestinal mucosa epithelial cell line (NCM460). NCM460 (INCELL, USA),	TiO 2 NPs elicit minimum biological responses from the intestinal cells.
Al-Rasheed et al. 2013 [145]	The aim of the study was to investigate the toxic impacts of titanium dioxide nanoparticles (TiO2- NPs) on rat kidneys and the possible prophylactic role of either quercetin or idebenone. TiO2-NPs were administered orally at either 600 mg or 1 g/kg body weight for 5 consecutive days.	Severity of cytotoxic impact on renal tissue was dose dependent with increasing serum renal functional biomarkers, elevation of inflammatory, more evident in rats that received the higher dose.

Murugadoss, et al., 2020 [75]	The focus of the study was to compare the magnitude of the toxicity/biological responses induced by small (SA) and large agglomerates (LA) of two TiO2 NPs with different primary particle sizes (17 and 117 nm). For in vitro testing, human bronchial epithelial (HBE), colon epithelial (Caco2) and monocytic (THP-1) cell lines were exposed to these suspensions for 24h; In vivo, C57BL/6JRj mice were exposed via oropharyngeal aspiration or oral gavage to TiO(2) suspensions and, after 3 days WTS-1 and LDH assays were used.	Both TiO2 NPs were not cytotoxic (24 h exposure) even in their most dispersed state.
Chakrabarti, et al., 2019 [104]	In vitro and in vivo cytotoxicity. Cell cycle and apoptosis due to TiO2-NPs were evaluated using resazurin assay and flow cytometric techniques. In vitro analysis was performed at the concentration range of 10–100 mg/mL using murine. RAW 264.7 cells. In vivo experiments were conducted on Albino mice (M/F) by exposing them to 200 and 500 mg/kg TiO2-NPs for 90 days.	The cell viability percentage decreased significantly in a concentration-dependent manner in TiO2-NPs treated RAW 264.7. As the concentrations of NPs were increased, cells experienced more oxidative damage and resided in the G2/M phase of the cell cycle. More cells at G2/M indicated apoptosis of the damaged cells, which in turn reveal genotoxicological impact of TiO2-NPs.
Mi-Rae et al., 2016 [45]	In vivo solubility, oral absorption, tissue distribution, and excretion kinetics of food grade TiO2 (f-TiO2) NPs were evaluated following a single-dose oral administration to rats and were compared to those of general grade TiO2 (g-TiO2) NPs. The intestinal transport pathway was also assessed using 3-D culture systems.	Overall absorptions of f-TiO2 and g-TiO2 were less than 0.8%, and most of the particles were directly eliminated through the feces, suggesting low toxicity potential of TiO2 NPs.
Brun et al 2014 [41]	To evaluate by in vitro models of gut epithelium (a monoculture of Caco-2 cells (regular epithelium), a co-culture of Caco-2 and HT29-MTX cells (mucus-secreting regular epithelium) and a coculture of Caco-2 and RajiB cells) as well as in vivo studies the impact and translocation of NPs through the gut epithelium.	TiO2-NPs would possibly translocate through both the regular epithelium lining the ileum and through Peyer's patches, would induce epithelium impairment, and would persist in gut cells where they would possibly induce chronic damage.
Morgan et al.	The objective of the study was to examine whether Tiron plays a modulatory role against apoptotic damage induced	TiO2 NPs significantly upregulated the proapoptotic Bax gene and downregulated the antiapoptotic Bcl-2 gene.

2018 [231]	by TiO2 NPs in rat livers. The nano-anatase TiO2 NPs were administered at an oral dose of 100 mg/kg/day for 60 days,	
McCraken et al., 2013 [99]	TiO2-NPs (21 nm) behavior in simulated digestion media and their interaction with intestinal epithelial cell line C2BBe1, a clone of Caco-2 cells.	TiO2-NPs exposed to the simulated digestion media exhibited mild toxicity based on decrease of mitochondrial activity. Nevertheless, they are internalized by the epithelial cells as nanoparticles and may subsequently enter the circulation and migrate to other parts of the body with unknown toxic effects.
Sycheva, et al., 2011 [176]	Toxicity of titanium dioxide were studied in a mouse model by oral gavage with titanium dioxide particles (microsized, TDM, 160 nm; nanosized, TDN, 33 nm) in doses of 40, 200 and 1000 mg/kg bw, daily for seven days.	The cytotoxic effect of TDN became manifest in the form of a significantly increased mitotic index in forestomach and colon epithelia (at the 40-mg/kg bw dose), and the frequency of round spermatids with two and more nuclei.
Chen et al 2019 [94]	This study aimed to illustrate the hepatotoxicity induced by TiO2 NPs and the underlying mechanisms. Rats were administered with TiO2 NPs (29 nm) orally at exposure doses of 0, 2, 10, 50 mg/kg daily for 90 days.	TiO2 NPs could induce slight hepatotoxicity at a dose of 50 mg/kg after long-term oral exposure.
