# Supplementary material for: Adverse Outcome Pathways Associated with the Ingestion of Titanium Dioxide Nanoparticles—A Systematic Review
Source: Nanomaterials (Basel). 2022 Sep 21;12(19):3275. doi: 10.3390/nano12193275 (PMC9565478; doi:10.3390/nano12193275)
Supplement: Supplementary file 1 [file nanomaterials-12-03275-s001.zip › Table S5.pdf]

Table S5 – Studies reporting the genotoxicity of TiO2-NPs in intestinal context.

Reference	Study description	Main findings concerning genotoxicity
Jensen et al. 2019 [73]	Assessment of gastrointestinal tight junction and systemic genotoxic effects in rats following exposure to E171 for 10 weeks by oral gavage once a week	Unaltered levels of oxidatively damaged DNA in the liver and lung and no changes in the DNA repair activity of oxidatively damaged DNA in the lung.
Proquin, et al., 2017 [85]	E171 exposure of human Caco-2 and HCT116 cells to analyze ROS formation and DNA damage and to investigate the contribution of the separate (MPs) and nano-sized (NPs) fractions to these effects.	The capacity for ROS generation in a cell-free environment was highest for E171, followed by NPs and MPs. Only MPs induced ROS formation in exposed Caco-2 cells. E171, MPs and NPs all induced single-strand DNA breaks. Chromosome damage was shown to be induced by E171, as tested with the micronucleus assay in HCT116 cells.
Gerloff, et al., 2012 [87]	Five TiO2-samples were analyzed for purity, crystallinity, primary particle size, SSA, zeta potential, and aggregation/agglomeration. The cytotoxicity, oxidative stress, and DNA damage was evaluated in human intestinal Caco-2 cells.	Only anatase-rutile containing samples, in contrast to the pure anatase samples, induced significant LDH leakage or mild DNA damage (Fpg-comet assay).
El-Din, et al., 2019 [234]	Investigate if curcumin ameliorates TiO2NP-induced cardiotoxic and genotoxic effects in adult male albino rats exposed by oral gavage once daily for 90 days	Comet assay results confirmed TiO2NP-related DNA damage. All these changes are partially mitigated in rats treated with both curcumin and TiO2-NPs.
Brown, et al., 2019 [88]	ToxTracker assay (mechanism-based reporter assay based on embryonic stem cells that uses GFP-tagged biomarkers for detection of DNA damage, oxidative stress and general cellular stress) used to evaluate food-grade TiO2 (E171)) and compared to tissue relevant cell types.	E171 to be the most cytotoxic, decreased intracellular glutathione and the most significant with regards to genotoxic effects. The ToxTracker data showed comparability to conventional toxicity and oxidative stress assays; however, some discrepancies were evident between the findings from ToxTracker and the comet assay.
Manivannan, et al., 2019 [167]	Swiss albino male mice were gavaged TiO2-NP at sub-acute concentration (0.2, 0.4 and 0.8 mg/kg body weight) over a period of 28 days	TiO2-NP administered was of rutile form and chromosomal aberration assay showed that TiO2-NP was genotoxic and cytotoxic. DNA damage evaluation by comet assay confirmed that long term exposure to TiO2-NP at low concentrations can induce genotoxicity systemically in organs, such as liver, spleen, and thymus cells. Structural chromosomal aberration test from bone marrow cells revealed the clastogenicity of TiO2-NP at sub chronic low concentrations.
Gerloff, et al., 2009 [116]	Cytotoxic and DNA-damaging effects of several types of nanoparticles and fine particles relevant as food additives (TiO2 and SiO2) or for food	DNA damage by TiO2 was found to depend on sample processing conditions. Interestingly, application of different TiO2 and ZnO

	packaging (ZnO and MgO) as well as carbon black on human intestinal Caco-2 cells	particles revealed no relation between particle surface area and DNA damage.
Hashem, et al., 2020 [235]	Effect of oral exposure of male Wistar rats to two doses of TiO2 (20 or 40 mg/kg b.wt.) through oral gavage once daily for 90 consecutive days on the blood components, immunity, cytotoxic, and genotoxic indicators.	A significant increase in the comet variables was recorded in response to the exposure of rats to the increasing level of TiO2 at both levels.
Mohamed, et al., 2015 [162]	Nano-TiO2 induced genotoxicity and possible induction of chronic gastritis in mice orally administered 5, 50 or 500 mg/kg body weight nano-TiO2 for five days, and sacrificed 24 h or one or two weeks after the last treatment	The administration of nano-TiO2 resulted in persistent apoptotic DNA fragmentation and mutations in p53 exons (5-8).
Kermanizadeh, et al., 2012 [220]	Effects of five titanium dioxide (TiO2) on oxidative responses of C3A cell line as a model	Following 4 hr exposure of the C3A cells to sub-lethal levels of the NMs, the largest amount of DNA damage was induced by two of the TiO2 samples (7 nm and the positively charged 10 nm particles).
Dekanski, et al., 2018 [236]	Acute toxicity of surface-modified TiO2 nanoparticles (NPs) with caffeic acid (CA) was compared with those of its separate constituents (free CA and bare TiO2 NPs) upon their oral administration in laboratory mice.	Acute toxicity signs, including biochemical alterations and extensive histopathological changes in the liver tissue of mice were detected 14 days after oral administration of bare TiO2 NPs.<br>Both, bare and surface-modified TiO2 NPs did not display a DNA damaging effect in a time frame of 24 h upon their oral administration in mice.
Grissa, et al., 2015[175]	Investigated the hematological effects and genotoxicity of anatase TiO2 NPs following sub-chronic oral gavage treatment. Wistar rats were treated with anatase TiO2 NPs by intragastric administration for 60 days	The micronucleus test revealed damage to chromosomes in rat bone marrow at 100 and 200 mg/kg bw; the comet assay showed significant DNA damage at the same doses.
Dorier, et al., 2019 [201]	In vitro impact of the food additive E171, i.e. TiO2, and of TiO2 nanoparticles, on a co-culture of Caco-2 and HT29-MTX cells; Alkaline and Fpg-modified comet assay and 8-oxo-dGuo measurement by HPLC-MS/MS; mRNA expression of genes involved in ROS regulation, DNA repair via base-excision repair, and endoplasmic reticulum stress was assessed by RT-qPCR	Increased intracellular ROS levels, but did not impair cell viability and did not cause any oxidative damage to DNA. Only minor changes in mRNA expression were detected. Altogether, this shows that E171 food additive and TiO(2) nanoparticles only produce minor effects to this in vitro intestinal cell model.
Teubl, et al., 2015 [96]	Biological impact of hydrophilic (NM 103, rutile, 20 nm) and hydrophobic (NM 104, rutile, 20 nm) TiO2 particles in buccal mucosa cells and HPRT gene mutation assay in V79 cells	Although cell viability/membrane integrity was not affected negatively, screening assays demonstrated that NM 104 particles showed a higher potential to decrease the physiological mitochondrial membrane potential than NM 103, resulting in a pronounced generation of reactive oxygen species. No effect on gene mutation induction.
Chen, et al., 2014 [173]	In vivo study, the adult male Sprague-Dawley rats were exposed to anatase TiO2 NPs (75 +/- 15 nm) through intragastric administration at 0, 10, 50 and 200 mg/kg body weight every day for 30 days	In vivo, TiO2 NPs could induce DNA double strand breaks (gamma-H(2)AX assay) in bone marrow cells. However, the micronucleus test

	In vitro study, Chinese hamster lung fibroblasts (V79 cells) were exposed to TiO2 NPs at the dose of 0, 5, 10, 20, 50 and 100 µg/mL.	did not show damage to chromosomes or mitotic apparatus observably in rat bone marrow cells.<br>In vitro, significant DNA damage was only observed at the concentration of 100 µg/mL after 24 h treatment using the comet assay. Gene mutation was observed at the concentration of 20 and 100 µg/mL after 2h treatment using hypoxanthine-guanine phosphoribosyl transferase (HPRT) gene mutation assay.
Shrestha, et al., 2016 [120]	Four kinds of TiO2 nanorods (TiO2 NRs) were used to study their interaction with rat bone marrow stem cells (MSCs).	The genotoxicity of the TiO2 NRs was neglectable at tested concentrations, studied by the comet assay.
Shukla, et al., 2014 [76]	Mice were exposed orally for 14 consecutive days to anatase TiO2	Significant oxidative DNA damage was observed in liver cells, which could be attributed to oxidative stress.
Grissa et al., 2017 [141]	Rats were given rosemary extract via intragastric administration 1 h before the intragastric administration of 100 mg/kg/day TiO2 (10 nm) for 60 days.	TiO2 NPs significantly increased serum cholesterol, glucose, and triglyceride levels of rats. They also induced significant oxidative stress and inflammation and caused DNA damage in peripheral blood leukocytes. The rosemary extract appears to have a significant protective effect.
Donner, et al., 2016 [157]	Six pigment-grade (pg) or ultrafine (uf)/nanoscale (anatase and/or rutile) titanium dioxide (TiO2) particulates were evaluated for in vivo genotoxicity (OECD 474 Guidelines) in male and female rats by two different laboratories; after single oral gavage doses of 500, 1000 or 2000 mg/kg body weight (bw) the materials were assessed for induction of micronuclei and toxicity in bone marrow by analyzing peripheral blood reticulocytes (RETs) by flow cytometry.	For each of the six tests, there were no biologically or toxicologically relevant increases in the micronudeated RET frequency in any TiO2 exposed group at either time point at any dose level. In addition, there was a lack of biologically relevant decreases in %RETs among total erythrocytes. All six TiO2 test substances were negative for in vivo genotoxicity effects. One pigment grade and one ultrafine material each were evaluated for potential systemic exposure/uptake from the gastrointestinal tract by analysis of TiO2 into blood and liver. No significant increases in TiO2 over controls were measured in blood (48 or 72 h) or liver (72 h) following exposures to 2000 mg/kg bw TiO2.
Azim et al., 2015 [78]	Nano-anatase TiO2 (21 nm) was administered (150 mg/kg/day) for 2 weeks followed by the antioxidants idebenone, camosine and vitamin E either alone or in combination for 1 month.	TiO2 effectively activated caspase-3 and caused liver DNA damage. Oral administration of idebenone (200 mg/kg), carnosine (200 mg/kg) and vitamin E (100 mg/kg) alleviated the hazards of TiO2 NPs with the combination regimen showing a relatively higher effect.
Botelho, et al., 2014 [178]	DNA damage by Comet assay on nanoparticle-treated AGS human gastric epithelial cell line in comparison to controls.	AGS cells show an increase in tail intensity in 150 mg/mL of TiO2 nanoparticles.<br>(Sigma)-treated cells compared to control.

Jalili, et al., 2018 [86]	Cytotoxic and genotoxic effects of two rutile TiO2 NMs, differing in surface coating, NM103 (hydrophobic) and NM104 (hydrophilic), on intestinal and hepatic cell models, following 3 or 24 h treatments with concentrations of TiO2 NMs from 1.2 to 80 µg/cm2	Although TEM demonstrated the presence of the two TiO2 NMs within the cytoplasm, no significant cytotoxic or genotoxic were observed in either cell model.
Bettini, et al., 2017 [3]	Rats orally exposed for one week to E171 at human relevant levels or NM 105 (anatase-rutile)	Titanium was detected in the immune cells of Peyer's patches (PP) as observed with the TiO2-NP model NM-105. Dendritic cell frequency increased in PP regardless of the TiO2 treatment, while regulatory T cells involved in dampening inflammatory responses decreased with E171 only, an effect still observed after 100 days of treatment. In all TiO2-treated rats, stimulation of immune cells isolated from PP showed a decrease in Thelper (Th)-1 IFN-gamma secretion, while splenic Th1/Th17
Morgan, et al., 2017 [56]	adult male albino rats exposed to TiO2 at 100 mg/kg/day orally for 8 weeks	The results revealed high incidence of micronucleated RBCs, elevated oxidative stress parameters and up regulation of Testin gene.
Dorier, et al., 2017 [81]	Cells were exposed to E171 and two different types of TiO2-NPs, either acutely (6-48 h) or repeatedly (three times a week for 3 weeks, using two in vitro models: (i) a monoculture of differentiated Caco-2 cells and (ii) a coculture of Caco-2 with HT29-MTX mucus-secreting cells	Results confirm that E171 damaged these cells, and that the main mechanism of toxicity was oxidation effects. Oxidative damage to DNA was detected in exposed cells, proving that E171 effectively induces oxidative stress; however, no endoplasmic reticulum stress was detected.
Mohamed, et al., 2016 [237]	Male Swiss Webster mice were orally administered TiO(2)NPs (500 mg/kg b.w.) daily for five consecutive days and then animals were sacrificed at 24 h, 7 days, or 14 days after the last treatment.	Comet assay revealed the apoptotic DNA fragmentation, while PCR-SSCP pattern and direct sequencing showed point mutation of Presenilin 1 gene at exon 5, gene linked to inherited forms of Alzheimer's disease. Therefore, from these findings, the present study concluded that TiO2 NPs is genotoxic and mutagenic to brain tissue which in turn might lead to Alzheimer's disease incidence.
Ali, et al., 2019 [77]	The effect of five days oral administration of TiO(2)NPs (21 and 80 nm) with different doses (50, 250 and 500 mg/kg body weight) was assessed in mice	The results revealed drastic alterations in all the measured parameters and showed positive correlation with the gradual dose increment. In addition, the smaller particle size of TiO2NPS (21 nm) had more adverse effects in all the selected biochemical parameters, genetic aberrations and histological investigations.
Kurzawa-Zegota, et al., 2017 [179]	The cytotoxic and genotoxic potential of TiO2 NPs was examined in peripheral blood lymphocytes in polyposis coli and colon cancer patients as well as healthy individuals. Cells from donors were exposed to nanoparticle concentrations ranging from 10 to 80 mu g/ml.	Concentration dependent effects of TiO2 NPs in both patient groups and healthy individuals were observed in the Comet assay, when OTM and % Tail DNA parameters were examined. Also the frequency of micronuclei (MN) in binucleated cells was increased in a

		concentration-dependent manner. Experiments revealed that polyposis coli and colon cancer patients had a higher level of DNA damage in the Comet assay and a higher number of MN than healthy individuals.
Elnagar, et al., 2018 [149]	Adult male albino rats treated with 1200 mg/kg TiO2 nanoparticles	TiO2 resulted in DNA damage (comet assay).
Schneider, et al., 2017 [107]	Toxicological behavior of metal nanoparticles (gold, silver) and metal oxide nanoparticles (copper oxide, zinc oxide, titanium dioxide) in vitro in human colorectal adenocarcinoma cells (HT29).	Genotoxic effects after nanoparticle treatment were not observed for the tested concentrations.
Agnihothram, et al., 2016 [122]	Effect of anatase TiO2 NPs on NoroVirus replication and host response during virus infection, using murine norovirus (MNV-1) infection of RAW 264.7 macrophages	No genotoxic insults due to anatase TiO2 NPs alone or to their presence during MNV-1 infection were detected.
Martins, et al., 2017 [59]	Distribution, redox parameters, and genotoxic effects in male Wistar rats that were treated with TiO2NP exposed via oral gavage to either sodium citrate buffer (vehicle), 0.5 mg/kg/day TiO2NP, 0.5 mg/kg/day AgNP or a mixture of TiO2NPs and AgNPs; Exposure 45 days after which rats were sacrificed.	The genotoxicity was analyzed using the comet assay in peripheral blood and liver cells. The individual or co-exposure to TiO2NP and AgNP did not markedly induce genotoxicity in blood or liver cells. Data showed that TiO2NP did not produce significant oxidative stress or genotoxicity in rats at the dose used in this study while the same dose level of AgNPs resulted in oxidative stress, but no noticeable adverse genotoxic effects.
Murugadoss, et al., 2020 [75]	Tested two TiO(2) NPs with different primary sizes (17 and 117,Äânm) and prepared ad-hoc suspensions composed of small or large agglomerates with similar dispersion medium composition. For in vitro testing, human bronchial epithelial (HBE), colon epithelial (Caco2) and monocytic (THP-1) cell lines were exposed to these suspensions for 24h;<br>In vivo, C57BL/6JRj mice were exposed via oropharyngeal aspiration or oral gavage to TiO(2) suspensions and, after 3 days	In vitro large agglomerates of TiO(2) induced stronger responses than small agglomerates for glutathione depletion, IL-8 and IL-1Œ≤ increase, and DNA damage in THP-1, while no effect of agglomeration was observed with 117nm TiO(2).<br>In vivo we observed that large agglomerates of 117nm TiO2 induced higher pulmonary responses in aspirated mice and blood DNA damage in gavaged mice compared to small agglomerates.
Chakrabarti, et al., 2019 [104]	in vitro and in vivo genotoxic endpoints were estimated by means of comet, micronucleus (MN), and chromosomal aberration (CA) assays; In vitro analysis was performed at the concentration range of 10-100 ug/mL using murine RAW 264.7 cells.<br>In vivo experiments were conducted on Albino mice (M/F) by exposing them to 200 and 500 mg/kg TiO2-NPs for 90 days.	Increased comet scores such as tail length, % DNA in tail, tail moment, and olive moment were also observed with the higher doses of TiO2-NPs in vitro and in vivo comet assays. Finally, the in vivo MN and CA assays revealed the formation of MN and chromosomal breakage following the exposure to TiO2-NPs.
Sycheva, et al., 2011 [176]	Genotoxicity and carcinogenicity of titanium dioxide were studied in a mouse model; CBAB6F1 mice by oral gavage with titanium dioxide particles	TDM induced DNA-damage and micronuclei in bone-marrow cells and TDN induced DNA-damage in the cells of bone marrow and liver.

	(microsized, TDM, 160 nm; nanosized, TDN, 33 nm) in doses of 40, 200 and 1000 mg/kg bw, daily for seven days.	TDM and TDN increased the mitotic index in forestomach and colon epithelia, the frequency of spermatids with two and more nuclei, and apoptosis in forestomach (only TDN) and testis.
