# Supplementary material for: Adverse Outcome Pathways Associated with the Ingestion of Titanium Dioxide Nanoparticles—A Systematic Review
Source: Nanomaterials (Basel). 2022 Sep 21;12(19):3275. doi: 10.3390/nano12193275 (PMC9565478; doi:10.3390/nano12193275)
Supplement: Supplementary file 1 [file nanomaterials-12-03275-s001.zip › Table S6.pdf]

Table S6: Studies reporting modifications of biochemical parameters after exposure to of TiO2-NPs.

Reference	Animals	Biological sample	Biochemical Parameters	Conclusions
**Liver function**				
Wang et al. (2007) [184]	CD-1 (ICR) mice	Blood (Serum)	ALP, ALT, AST, TBIL	No significant differences for ALP, AST and TBIL.<br>ALT and ratio ALT/AST significantly higher in TiO2 25 nm group indicating liver toxicity.
Bu et al. (2010) [40]	Wistar rats	Blood (Serum)	ALT, AST, CHOL, TBIL, TG	TiO2 significantly increases AST serum levels.<br>Liver damage may occur after repeated oral administration of TiO2 NPs.
Chen et al. (2015) [185]	Sprague-Dawley rats (3 weeks old)	Blood (Serum)	ALB, GLB, ALB/GLB ratio, ALT, AST, ALT/AST ratio, TBIL	Exposure to TiO2 NPs (10 and 50 mg/kg bw/day) significantly increased GLB serum levels and decreased TBIL serum levels.<br>The co-exposure to glucose caused additionally the significant decrease of ALT, AST and ALB/GLB ratio.<br>Possible liver damage.
Azim et al. (2015) [78]	Albino mice (male)	Blood (Serum)	ALT, AST	Exposure to TiO2 NPs increased significantly the ALT and AST serum levels, compared to the control group.<br>Treatment with idebenone, carnosine, vitamin E and their combination exhibited a significant reduction in liver function enzyme activities as compared to TiO2 NPs group. This reduction reflected the hepatoprotective effect of these agents.
Natarajan et al. (2015) [119]	Sprague-Dawley rats (male)	Primary rat hepatocytes	ALB, BUN	Exposure to TiO2 demonstrated a concentration and type dependent loss in urea and albumin synthesis function of hepatocytes.

Yang et al. (2017) [56]	C57/BL6 mice (5-7 week old)	Blood (Serum)	ALP, ALT, AST, DBIL, IBIL, TBA	Exposure to 500 mg/kg/day of TIO2 increased significantly the serum bilirubin in a dose dependent manner.<br>No modifications were found for ALP, ALT, ASR and TBA, suggesting that bilirubin could be used as an early toxicity biomarker of nanomaterials.
Abbasi-Oshaghi et al. (2019) [72]	Wistar rats (male, 8 weeks old)	Blood (Serum)	ALP, ALT, AST, LDH, TBIL	Exposure to TiO2 NP significantly increased the ALP, ALT, AST and LDH serum levels, indicating liver damage.
Moradi et al. (2019) [143]	Wistar rats (male, 6–8 weeks old)	Blood (Serum)	ALP, ALT, AST, LDH	Exposure to TiO2 NP increased significantly the ALT, AST, ALP, and LDH serum levels, indicating liver damage.<br>A protective effect was demonstrated for vitamin A and E by its membrane stabilizing and antioxidant activity. The combination of both vitamins had a significantly greater effect on prevention of liver enzyme leakage than vitamin A which could demonstrate the better efficiency of vitamin E to scavenge free radicals.
**Cardiac damage**				
Wang et al. (2007) [184]	CD-1 (ICR) mice	Blood (Serum)	CK, alpha-HBDH, LDH	Nanosized particles induced more severe myocardial damage than fine particles. For the TiO2 80 nm group (female mice), LDH and HBDH were higher than the control group and fine group ($p<0.05$).
Chen et al. (2015) [185]	Sprague-Dawley rats (3 weeks old)	Blood (Serum)	CK, HBDH, LDH	Exposure to TiO2 NPs (10 and 50 mg/kg bw/day) significantly decreases CK in females and LDH and HBDH in males during the 90 days exposure. The co-exposure to glucose caused the same effects at 30 and 90 days of exposure. Possible cardiac damage.
Bu et al. (2010) [40]	Wistar rats	Blood (Serum)	CK, LDH	The exposure to 0.4 and 1 g/Kg bw/day of TiO2 significantly increase CK and LDH serum levels. Data suggest that cardiac damage may occur after repeated oral

				administration of TiO2 NPs.
**Nephrotoxicity**				
Wang et al. (2007) [184]	CD-1 (ICR) mice	Blood (Serum)	BUN, CREA, UA	Exposure to 5 g/kg bw of TiO2 particles, and due to the small size and difficult clearance of TiO2, the retention of different sized TiO2 particles in vivo induced the damage of kidneys.<br>For the TiO2 25 nm group (female mice), BUN was significantly increased when compared to the control group and fine particles group.
Chen et al. (2015) [185]	Sprague-Dawley rats (3 weeks old)	Blood (Serum)	BUN, CREA	Exposure to TiO2 NPs (10 and 50 mg/kg bw/day) significantly increased BUN serum levels and decreased CREA serum levels in male rats. The co-exposure to glucose caused the same effects at 30 and 90 days of exposure.<br>Possible kidney damage.
Abbasi-Oshaghi et al. (2019) [72]	Wistar rats (male, 8 weeks old)	Blood (Serum)	BUN, CREA	Exposure to TiO2 NP (50 and 100 mg/kg) significantly increased the BUN and CREA serum levels, indicating kidney damage.
**Hematological effects**				
Chen et al. (2015) [185]	Sprague-Dawley rats (3 weeks old)	Blood (Plasma EDTA)	WBC, LYM, MOD, GRN, LYM%, MOD%, GRN%, RBC, HGB, HCT, MCV, MCH, MCHC,	Exposure to TiO2 NPs significantly increases hematological parameters being attributed to the activation of immune function and inflammatory response (for parameters related with WBC) and to an adaptive body response to the toxic effects of TiO2 NPs (for parameters related with RBC).

			RDW, PLT, PCT, MPV, PDW	
		Blood (Plasma Citrate)	APTT, FIB, PT	No significant alterations in coagulation parameters.
Bu et al. (2010) [40]	Wistar rats	Blood (Plasma EDTA)	WBC, NEU, LYM, MON, RBC, HGB, PLT	The exposure to 1 g/Kg bw/day of TiO2 significantly increases WBC, LYM and MON cell counts.<br>Data suggest that inflammation may be induced after repeated oral administration of TiO2 NPs.

Lipid metabolism

Chen et al (2020) [188]	Sprague-Dawley rats (3 weeks old)	Blood (Serum)	LDL-C, HDL-C, TCHO, TG	Exposure to TiO2 NPs (50 mg/kg) could have an impact on lipid metabolism in rats.<br>The level of TG significantly decreased.<br>The lipidomic signature changed significantly in the serum of rats treated with TiO2 NPs.

Glucose metabolism

Chen et al (2018) [189]	Sprague-Dawley rats (3 weeks old)	Blood (Serum)	GLU, HbA1c, GSP, INS, C-PEP, GLUC	Results of GLU, GSP and HbA1c over time showed that oral exposure to TiO2 NPs could cause a slight and temporary hypoglycemic effect on rats, which appeared within subacute oral exposure, but disappeared when oral exposure was subchronic.<br>Secretion of insulin and glucagon were unaffected. The low level of blood insulin

				was most likely due to the increased insulin metabolism in the liver to compensate for the hypoglycemic effect caused by subacute exposure of TiO2 NPs and to promote the recovery of blood glucose homeostasis.
		Blood (total blood, tail)	GLU	Exposure to 10 mg/kg bw/day TiO2 NP during 30 days showed an increased postprandial blood glucose concentration, thus indicating that TiO2 NPs could cause reduced glucose tolerance.

Prostatic and testicular toxicity

Shahin et al (2017) [114]	Wistar rats (male)	Blood (Serum)	E2, FSH, LH, PAP, PSA, TST	Exposure to TiO2 NP for 2 and 3 weeks showed a significant increase in PSA and PAP serum levels. This effect on PSA serum levels was significantly attenuated by morin co-treatment. Morin co-treatment showed no effect on increased PAP serum levels.<br>Exposure to TiO2 NP for 1, 2 and 3 weeks showed a significant decrease in TST serum levels that were only significantly attenuated by morin co-treatment in week 1.<br>Exposure to TiO2 NP for 2 and 3 weeks showed a significant increase in E2, LH and FSH serum levels and ACP testicular activity, which were significantly attenuated by morin co-treatment.<br>Exposure to TiO2 NP for 1, 2 and 3 weeks showed a significant increase in GGT testicular levels that were significantly attenuated by morin co-treatment.<br>Significant ameliorative potential of morin against nTiO2-induced prostatic and testicular toxicity was determined, with such beneficial roles of morin probably being mediated by redox regulatory, anti-inflammatory and anti-apoptotic mechanisms.
		Testis and Prostate	ACP, GGT	

Table S6 Footnote: Activated partial thromboplastin time (APTT), alanine aminotransferase (ALT), albumin (ALB), alkaline phosphatase (ALP), alpha-hydroxybutyrate dehydrogenase (HBDH), aspartate aminotransferase (AST), C-peptide (C-PEP), creatine kinase (CK), creatinine (CREA), estradiol (E2), fibrinogen (FIB), free testosterone (TST), follicle stimulating hormone (FSH), gama-glutamil-transferase (GGT), glucose (GLU), glycated hemoglobin (HbA1c), glycated serum protein (GSP), globulin (GLB), glucagon levels (GLUC), granulocytes (GRN), hemoglobin concentration (HGB), hematocrit (HCT), high density lipoprotein cholesterol (HDL-C), indirect bilirrubin (IBIL), insulin (INS), lactate dehydrogenase (LDH), low density lipoprotein cholesterol (LDL-C), luteinizing hormone (LH), lymphocytes (LYM), monocytes (MOD), percentage of lymphocytes (LYM%), percentage of monocytes (MOD%) and percentage of granulocytes (GRN%), red blood cells count (RBC), mean corpuscular volume (MCV), mean corpuscular hemoglobin (MCH), mean corpuscular hemoglobin concentration (MCHC), mean platelet volume (MPV), platelet count (PLT), platelet hematocrit (PCT), platelet distribution width (PDW), prostatic acid phosphatase (PAP), prostate specific antigen (PSA), prothrombin time (PT), red blood cell volume distribution width (RDW), total bilirubin (TBIL), total bile acid (TBA), total cholesterol (TCHO), triglycerides (TG), urea (BUN), uric acid (UA), white blood cells count (WBC)
